# The *Mycoplasma* spp. ‘Releasome’: A New Concept for a Long-Known Phenomenon

**DOI:** 10.3389/fmicb.2022.853440

**Published:** 2022-04-15

**Authors:** Patrice Gaurivaud, Florence Tardy

**Affiliations:** Anses, Laboratoire de Lyon, VetAgro Sup, UMR Mycoplasmoses Animales, Université de Lyon, Lyon, France

**Keywords:** secretome, polysaccharide, *Mycoplasma*, extracellular vesicles, exoproteome

## Abstract

The bacterial secretome comprises polypeptides expressed at the cell surface or released into the extracellular environment as well as the corresponding secretion machineries. Despite their reduced coding capacities, *Mycoplasma* spp. are able to produce and release several components into their environment, including polypeptides, exopolysaccharides and extracellular vesicles. Technical difficulties in purifying these elements from the complex broth media used to grow mycoplasmas have recently been overcome by optimizing growth conditions and switching to chemically defined culture media. However, the secretion pathways responsible for the release of these structurally varied elements are still poorly described in mycoplasmas. We propose the use of the term ‘releasome,’ instead of secretome, to refer to molecules released by mycoplasmas into their environment. The aim of this review is to more precisely delineate the elements that should be considered part of the mycoplasmal releasome and their role in the interplay of mycoplasmas with host cells and tissues.

## Introduction

The term bacterial secretome was coined in the very beginning of the 21st century (Tjalsma et al., 2000) to describe “the components of machineries for protein secretion and the native secreted proteins,” with a primary focus on the secretion systems, i.e., machineries, involved in membrane translocation. Since then, the definition of the secretome has been regularly revised to focus more on the final localization of the secreted proteins and their potential distinct associated functions (Desvaux et al., 2009; Armengaud et al., 2012; Monteiro et al., 2021). The concept of the secretome now includes surface-exposed proteins (surface proteome), proteins released as free (not cell-attached) into the bacterial environment (exoproteome) and proteins embedded in extracellular vesicles (proteovesiculome) (Monteiro et al., 2021). The bacterial secretome plays a range of roles in interactions with the host (including adhesion, invasion, immune evasion and modulation), nutrient acquisition, and interactions between bacterial cells. Some of these interactions are positive, like biofilm formation, while others can be negative, like competition (Chagnot et al., 2013; Sharma et al., 2017; Tommassen and Arenas, 2017; Hernandez et al., 2020). Many of the proteins involved in virulence are found in the secretome, and characterizing these proteins is essential to understanding host-pathogen interactions and ultimately to developing a suitable disease control strategy (Dwivedi et al., 2016). However, although most of the extant literature has focused on secreted proteins, other polymers, like polysaccharides, are also exported by bacteria and fulfill different functions, depending on their final location, i.e., either cell-attached or not (Desvaux et al., 2009; Woodward and Naismith, 2016). Here we propose to (i) broaden the concept of the bacterial secretome to include non-polypeptide molecules like polysaccharides and more complex elements like extracellular vesicles (EV), and to (ii) rename this broader group of components *releasome* in order to take account of the gaps in knowledge about secretion machineries in some bacterial models (which is especially true for mycoplasmas) and to alter the focus exclusively to non-cell-attached elements released by living cells.

Polysaccharides are long-chain polymers composed of sugar units linked by glycosidic bonds. Extracellular polysaccharides either are attached to the cell and form a capsule or a slime layer around the cell, or are secreted as cell-free polysaccharides into the bacterium’s immediate environment. The generic term *exopolysaccharides* was proposed in 1972 to describe these two extracellular localizations (Sutherland, 1972). Many studies on exopolysaccharides fail to clearly specify their precise localization (free vs. cell-attached). However, it is essential to make a distinction between cell-linked and cell-free exopolysaccharides, as the two forms may play different roles in host interactions. The methodology used for purification provides clues about the type of exopolysaccharides under study: cell-linked polysaccharides are purified from washed cell pellets, while cell-free polysaccharides are purified from cell-free supernatants. In this review, for purposes of clarification, we use the acronym for cell-attached polysaccharides (CPS) that can form a capsule or a slime around the cell, and we use the term exopolysaccharides (EPS) strictly to refer to cell-free polysaccharides. The fact the composition and structure of the polysaccharide moiety might be identical with a single shared biosynthesis pathway (Woodward and Naismith, 2016) can make it difficult to distinguish between CPS and EPS: for instance, stresses can release CPS into the environment (Sutherland, 1985; Cerning, 1990; Llobet et al., 2008), and structures such as biofilms can make it difficult to assess cell attachment.

Extracellular vesicles are the most complex structures of the releasome, as they are composed of lipids, proteins, glycoconjugates and nucleic acids. Their composition reflects their biogenesis from the cell (Kim et al., 2015; Toyofuku et al., 2019). Bacterial EV are defined as non-replicative, membranous spherical structures with a size ranging from 20–400 nm that are secreted by viable cells (Deatherage et al., 2009; Toyofuku et al., 2019).

**FIGURE 1:**
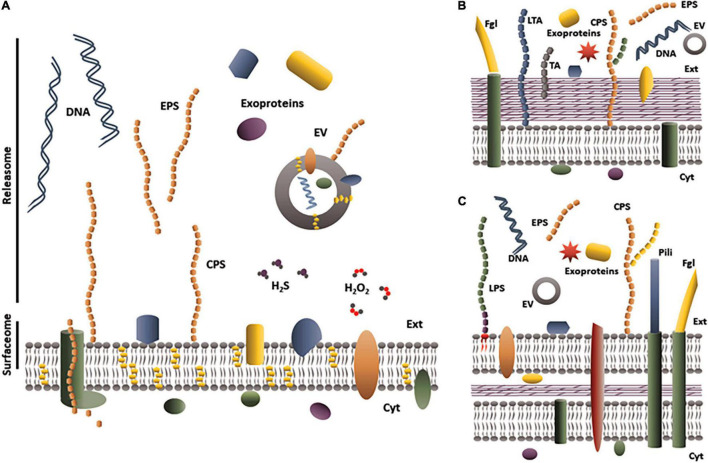
Schematic representation of the releasome of **(A)** *Mycoplasma* spp., **(B)** Gram-positive bacteria, and **(C)** Gram-negative bacteria. Mycoplasmas are only limited by a cholesterol (yellow hexagons)-rich phospholipid membrane. The cytoplasmic membrane of Gram-positive bacteria is surrounded by a thick peptidoglycan cell wall. In Gram-negative bacteria, the peptidoglycan layer is thin but is surrounded by a phospholipid outer membrane. EV, extracellular vesicles; EPS, exopolysaccharide; Cyt, cytoplasm; Ext, extracellular compartment; CPS, capsular polysaccharide; H_2_O_2_, hydrogen peroxide; H_2_S, hydrogen sulfide; LPS, lipopolysaccharide; LTA, lipoteichoic acid; TA, teichoic acid; Fgl, flagella; DNA, deoxyribonucleic acid.

Figure 1 summarizes all potential components included in the releasome to date, i.e., a subset of polypeptides (whatever the secretion pathways) plus polysaccharides, EV, nucleic acids and metabolites (such as H_2_O_2_ or H_2_S) released into the extracellular milieu. The releasome definition is particularly suitable for bacteria of the genus *Mycoplasma*, for which secretion systems are poorly known or poorly predictable *in silico* (Zubair et al., 2020a). Mycoplasmas, i.e., bacteria belonging to the genus *Mycoplasma* (*M.*), are small (300–800 nm diameter), wall-less bacteria with only a phospholipid, cholesterol-rich bilayer membrane surrounding the cytoplasm (Figure 1A, in contrast to panels B,C which show Gram+ or Gram– bacteria, respectively). Released molecules are ‘just’ translocated through the cytoplasmic membrane, which is a simpler situation than in the Gram+ and Gram− bacteria (Desvaux et al., 2009). Because of their small genome, coding for less than 1000 proteins, and the paucity of their metabolic pathways, mycoplasmas are considered the simplest bacteria able to replicate in an acellular medium (Razin et al., 1998). The first evidence of cell-free molecules released by mycoplasmas is historically related to the first culture (in 1898) of *M. mycoides* subsp. *mycoides*, the causal agent of contagious bovine pleuropneumonia (CBPP) (Nocard and Roux, 1898). The authors used a collodion bag containing broth inoculated with serous pulmonary fluid from a CBPP-diseased cow and placed into a peritoneal cavity of a rabbit. Mycoplasmas were able to survive and grow inside this non-permissive host because the collodion bag was permeable to host-released nutrients but offered protection against the rabbit immune system (Bove, 1999). Their work also found evidence that the collodion bag released a mycoplasma-produced component that induced necrosis in the surrounding tissues and might have contributed to cachexia in the rabbit (Lloyd, 1966). Other studies in the 1960s–1970s demonstrated some cell-free molecules released by mycoplasmas, such as polysaccharides or antigens recovered from the body fluids of CBPP-infected animals (Plackett et al., 1963; Gourlay, 1965), nanosized globular elements observed by microscopy in *M. pneumoniae* broth cultures (Eng and Froholm, 1971), and uncharacterized molecules that had cytotoxic activity in the supernatant of *M. bovigenitalium* cultures (Afshar, 1967). However, investigators at the time were unable to precisely identify the chemical structure of these elements, as they were unable to purify them from components of the complex growth medium or from body fluids. These experimental bottlenecks have recently been eased through various strategies that are covered in the first part of this review. In the second part, we propose an updated picture of the known composition and role of three main components of the releasome—exoproteins, EPS, and EV—in *Mycoplasma* spp. This concerns only components released into supernatants of mycoplasma growth medium alone or in interaction with host cells. Cellular invasion by mycoplasmas have been described for several species, including *M. hyopneumoniae* (Raymond et al., 2018b), *M. bovis* (Burki et al., 2015), *M. genitalium* (McGowin et al., 2009), *M. fermentans* (Yavlovich et al., 2004a), and *M. pneumoniae* (Yavlovich et al., 2004b) and the phenomenon is likely to be widespread. However, components released by mycoplasmas once in this cytoplasmic compartment of the host cell have not been studied thus far. Molecular mechanisms leading to the release of exoproteins, EPS and EV will not be covered here except for purpose of understanding the potential dual localization of molecules. The third and final section discusses the role of the mycoplasmal releasome.

## Experimental Bottlenecks to Characterizing the Releasome of Mycoplasmas *In Vitro*

**FIGURE 2:**
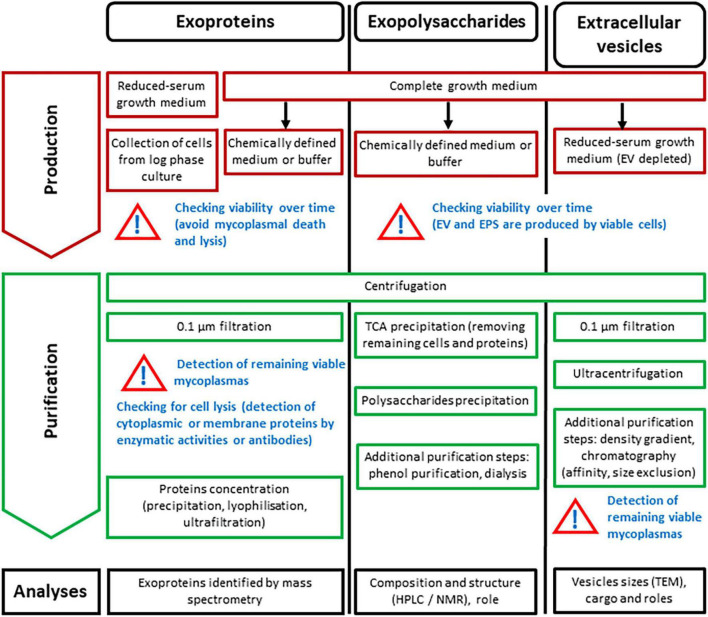
Strategies for *in vitro* production and purification, as well as analysis of exoproteins, exopolysaccharides and extracellular vesicles for characterization of the *Mycoplasma* releasome. Black arrows: switch to.

Molecules found free in a bacterial growth medium can have 3 distinct origins: (i) they could be brought by some complex components of the medium itself (such as serum, for instance), (ii) they could be released as a result of cell lysis during the different growth phases or purification processes, or (iii) they could be actively secreted or “simply” released (in the absence of known secretion machineries) by the bacterium. The releasome corresponds to the third category only, and one of the main experimental difficulties is to distinguish this category from the two others. Interference from medium components and from non-specific release of mycoplasma components (due to cell lysis in the different growth phases or harsh purification processes) varies between the different classes of molecules (proteins, polysaccharides, membrane vesicles) and the methodology used for their characterization (Figure 2). Exoproteins are usually identified by mass spectrometry, exopolysaccharides are identified by HLPC and NMR, and EV are first observed by TEM, and their composition is characterized using various biochemical techniques. *In vitro* characterization of the releasome necessitates a fine balance between placing mycoplasmas in the conditions where they actually release components (whether or not related to the total biomass produced) and finding experimental conditions that enable detection or purification of these components from the culture supernatant. The composition of the culture medium, the growth time before harvest, and the potential interspecies or interstrain diversity also need to be considered, as they can substantially influence the composition of the releasome (Bertin et al., 2015; Monteiro et al., 2021; Olaya-Abril et al., 2021).

### Exoproteome

Two recent papers have summarized the experimental bottlenecks and strategies for purification of the mycoplasmal exoproteome (Zubair et al., 2020a; Zhang et al., 2021). The authors pointed out that the high polypeptide concentrations in the growth media contributed by components such as serum dramatically jeopardizes the purification of proteins released by mycoplasmas, as is the case for exoproteins from eukaryotic cells that require serum for growth (Armengaud et al., 2012). Indeed, the 20% serum supplementation, usually used in mycoplasma medium, adds up to 12 g of proteins/L, which is dramatically more than the 2 mg of mycoplasma biomass/L reached by *M. hyopneumoniae* under optimized growth conditions (Hwang et al., 2010). A simple solution is to lower the relative concentrations of serum, as has been done to study the exoproteome of *M. hyopneumoniae*/*M. flocculare* (Paes et al., 2017) and *M. bovis* (Zubair et al., 2020b). However, such modifications of the growth medium dramatically affect the growth of mycoplasmas (Paes et al., 2017; Zubair et al., 2020b). Another strategy is to employ an ultrafiltration process to remove polypeptides from the growth medium prior to use. Voros et al. assessed the exoproteome of *M. capricolum* subsp. *capricolum* grown in a 10 kDa filtered complex medium (Voros et al., 2015). This process depletes proteins with a molecular weight greater than 10 kDa, but it comes at a risk of also removing lipids and cholesterol, which are essential for mycoplasma growth. However, the ultra-filtered medium nevertheless allowed residual growth of *M. capricolum* subsp. *capricolum* (Voros et al., 2009). Another method for bypassing this problem is to use a two-step approach (Figure 2), i.e., first, to produce a reasonable quantity of viable mycoplasma cells using complex growth media, and then to transfer the cells into a chemically defined medium with a reduced protein concentration (Ganter et al., 2019) or in an isotonic buffer like PBS (Zhang et al., 2021). Transferring mycoplasmas from a complex to a simpler medium requires centrifugation and washing steps that are associated with a risk of cell lysis and consequently release of cytoplasmic proteins. In a medium allowing growth (complete or serum-reduced), mycoplasmas can be harvested during the log phase, assuming that no cell lysis occurs. When mycoplasmas are incubated in a medium other than those allowing growth, the viability of the mycoplasmas over the incubation time needs to be checked, for instance by plating aliquots onto solid media and counting colonies (Ganter et al., 2019; Zhang et al., 2021; Figure 2).

Centrifugation of mycoplasma cells is an essential step in the process of purifying exoproteins from the broth supernatant (Figure 2). However, even at 15000 *g*, viable mycoplasmas may remain in the supernatant (Hopfe et al., 2004). Increasing centrifuge force to completely remove floating cells (Zubair et al., 2020b) might not be the best solution, as it could damage the cells and result in cell lysis (Razin et al., 1973). A preferred solution would be to filter the supernatant through a 0.1-μm filter before characterizing the exoproteome. While 0.2-μm filters can stop mycoplasma cells they do not completely remove all mycoplasma species (Gaurivaud et al., 2018). An agar plate count of viable mycoplasmas remaining in the supernatant is an easily performed control, but ensuring the total absence of viable mycoplasma cells is highly dependent on the test sample seeded on the agar plate. The control of potential cell lysis during purification could be ensured by detecting cytoplasmic or membrane proteins in supernatants using antibodies or assays for cytoplasmic enzymes such as lactate dehydrogenase (Voros et al., 2015) or hexokinase (Minion et al., 1993). However, caution is warranted when interpreting the results, as some moonlighting proteins expected to be cytoplasmic or membrane-bound can also be released into the extracellular environment as discussed below. For the same reason, the extracellular localization of a specific protein needs to be double-checked to confidently exclude any contamination by cell proteins during the exoproteome purification process. Protein localization is done either by detecting their enzymatic activities if feasible [e.g., extracellular nucleases; (Yamamoto et al., 2017)], or by using specific antibodies (Djordjevic et al., 2004; Zhang et al., 2016; Li et al., 2019). Such tests can be performed directly in complex medium and are less influenced by the contamination from cell lysis associated with transfer to a defined medium.

### Exopolysaccharides

As with the exoproteome, a complex growth medium is not suitable for mycoplasmal EPS purification (Bertin et al., 2013). Serum and yeast extracts contained in the growth medium contribute high concentrations of exogenous polysaccharides that hamper purification of the mycoplasmal polysaccharides. Because very low quantities of EPS are produced by mycoplasmas [a maximum of 50 mg/L for *M. mycoides* subsp. *mycoides* (Bertin et al., 2013), while *Klebsiella* or *Acinetobacter* spp. can produce up to 6 g/L (Bryan et al., 1986)], partial depletion of exogenous polysaccharides is not sufficient, so the alternative is to use a defined culture medium that is totally devoid of polysaccharides. The effort to develop serum-free defined media started in the 1960s (Tourtellotte et al., 1964) with the aim of determining mycoplasma nutrient requirements more precisely (e.g., preferred carbon sources) and studying their general metabolism [e.g., sugar biosynthesis pathways; (Yus et al., 2009; Jordan et al., 2013)]. However, although defined culture media enable control of substrate concentrations, they remain difficult to produce and use even today, and have not been optimized for all mycoplasma species. A simpler alternative consists of using serum-free eukaryotic cell culture media such as CMRL, which contains only glucose as a carbon source—a strategy we previously used to purify polysaccharides secreted by mycoplasmas belonging to the *M. mycoides* cluster (Bertin et al., 2013, 2015) and by *M. agalactiae* (Gaurivaud et al., 2016). In culture media used for eukaryotic cell growth, mycoplasmas may maintain a degree of viability, but are unable to grow. Therefore, to reach a satisfactory biomass, mycoplasmas have to be first grown in a complex medium, with cells pelleted and washed before being transferred into the defined eukaryotic cell medium (Figure 2). During incubation in CMRL, mycoplasmas remain viable and metabolically active for a period of time, with the duration dependent on the species. Polysaccharides are produced by viable mycoplasmal cells, and so a viability time–course has to be checked before purification of the polysaccharides (Figure 2). However, even if some mycoplasmas are lysed in the process, this does not result in non-specific release of CPS (Bertin et al., 2013). After incubation, mycoplasma cells are removed by centrifugation. A supplementary 0.1-μm filtration step is not essential, as the remaining cells and proteins in the supernatant are removed by trichloracetic acid precipitation (Figure 2). Finally, the polysaccharides are precipitated with acetone or ethanol. The level of purification can be increased by adding a further step, such as dialysis, to remove free hexoses, which interfere with the sugar composition measured by HPLC, and phenol treatment to remove biologically active small peptides (Totte et al., 2015).

Antibodies raised against polysaccharides are a good screening tool to help track the purification process or detect EPS in biological fluids (Bertin et al., 2015). However, they are not easily produced, as large amounts of purified polysaccharides conjugated to a carrier protein are required.

### Extracellular Vesicles

The ultracentrifugation step necessary to collect EV produced in a complex growth medium often results in an unwanted non-specific adsorption of albumin and immunoglobulins from the serum onto the surface of the vesicles, thus jeopardizing purification. The same difficulties are met during purification of exosomes from blood (Caradec et al., 2014). Reducing the serum and yeast extract concentrations is a good option, as it limits this ‘co-precipitation’ and also induces a nutritional stress conducive to vesicles formation and shedding (Klimentova and Stulik, 2015). This approach has been used for EV purification from *Mycoplasma* spp. and species of another close genus also belonging to the class *Mollicutes*, *Acholeplasma* (Chernov et al., 2011; Gaurivaud et al., 2018; Figure 2). Other stresses favoring EV production might be considered, such as iron deprivation through the use of chelators (Madsen et al., 2006; Martinez-Torro et al., 2020). Serum contains eukaryotic membrane vesicles that have to be removed before preparing the growth medium in order to ensure they are not co-purified them with the EV (Kornilov et al., 2018). *In vitro*, EV are only produced by viable mycoplasma cells, and we demonstrated previously that lysed, heat-killed cells do not produce non-specific EV-like vesicles (Gaurivaud et al., 2018). A viability control is therefore necessary during experiments to produce EV.

Before ultracentrifugation of the broth medium supernatant to collect the EV, a preliminary 0.1-μm filtration is advisable to remove small mycoplasma cells that could still be in suspension after elimination of the pelleted cells (Figure 2). However, this filtration step could also remove some of the large vesicles and hence select a sub-population of EV (Konoshenko et al., 2018), as there is a clear size overlap between small cells and larges vesicles (Gaurivaud et al., 2018).

Once pelleted, EV can be further purified using density gradients (sucrose, Optiprep™) or chromatography, as described for Gram-positive and Gram-negative bacterial vesicles (Kim et al., 2015; Klimentova and Stulik, 2015). This supplementary purification step has already been validated for *Acholeplasma laidlawii* (Chernov et al., 2014) and could be used for *Mycoplasma* spp., where it would help to remove any residual mycoplasma cells that might interfere with characterization of the polypeptides (by MS) and the DNA content (by PCR) of the purified EV.

## Known Elements of the Mycoplasmal Releasome

### Exoproteome

Mycoplasma exoproteomes have been a recent focus of research and these studies have benefited from the considerable developmental advances in other bacterial models (Zubair et al., 2020a). The first partial exoproteome was obtained in 2012 for *M. synoviae* (Rebollo Couto et al., 2012), followed by those of *M. hyopneumoniae*, *M. flocculare*, *M. bovis* and *M. capricolum* subsp. *capricolum* cultivated in axenic conditions (Voros et al., 2015; Paes et al., 2017; Zubair et al., 2020b; Zhang et al., 2021). Another study explored the exoproteome produced by swine mycoplasmas in interaction with their host cells (Leal Zimmer et al., 2019). However, relevant studies remain scarce and are not readily comparable because of the use of different methodological approaches (strains, culture conditions, purification steps). Because of these problems with comparability, we have focused only on exoproteins that have a specific, detectable function (Table 1).

**TABLE 1:** Non-exhaustive list of exoproteins with known functions released by *Mycoplasma* (sub)species.

Proteins	Identification	Species	Strains	Mnemonic/accession number	Culture conditions	Detection in extracellular environment	References
Nucleases	Ca^2+^/Mg^2+^ nuclease	*M. hyopneumoniae*	V11	Mhp597	Complete growth medium	Western blotting	Li et al., 2019
			7448	MHP7448_0580	Serum reduced medium	Exoproteome	Paes et al., 2017
			J	MHJ_0581	Infected cells (serum free medium)	Exoproteome	Leal Zimmer et al., 2019
	Endonuclease/exonuclease	*M. bovis*	HB0801	MBOV_RS02825	Complete growth medium	Western blotting	Zhang et al., 2016
	Mg^2+^ nuclease	*M. pneumoniae*	M129	MPN491	Complete growth medium	Zymography	Yamamoto et al., 2017
	Lipoprotein P40	*M. penetrans*	GTU-54-6A1	MYPE4380	Complete growth medium	Zymography	Bendjennat et al., 1997
	Superantigen	*M. arthritidis*	PG6	Marth_orf036	Complete growth medium	Lymphocytes proliferation	Atkin et al., 1994

Peptidases	S41 family peptidase	*M. mycoides* subsp. *capri*	95010	MMCAP2_0241	Complete growth medium	Casein hydrolysis	Ganter et al., 2019
		*M. capricolum* subsp. *capricolum*	Ckid	MCAP_0240	Complete growth medium	Casein hydrolysis	Ganter et al., 2019
	Putative peptidase DUF31	*M. bovirhinis*	MV5	MBVR141_0224	Complete growth medium	Casein hydrolysis	Ganter et al., 2019
	Oligoendopeptidase F	*M. capricolum* subsp. *capricolum*	Ckid	MCAP_0193	Serum reduced medium	Exoproteome	Voros et al., 2015
		*M. hyopneumoniae*	7448	AAZ53887.2	Infected cells (serum free medium)	Exoproteome	Leal Zimmer et al., 2019
		*M. flocculare*	ATCC 27716	ENX51111.1	Infected cells (serum free medium)	Exoproteome	Leal Zimmer et al., 2019
		*M. bovis*	HB0801	Mbov_0133	Serum reduced medium	Exoproteome	Zubair et al., 2020b
	Zinc metalloprotease, putative	*M. capricolum* subsp. *capricolum*	Ckid	MCAP_0804	Serum reduced medium	Exoproteome	Voros et al., 2015
	Dipeptidase, putative	*M. capricolum* subsp. *capricolum*	Ckid	MCAP_0420	Serum reduced medium	Exoproteome	Voros et al., 2015
	Cytosol aminopeptidase	*M. capricolum* subsp. *capricolum*	Ckid	MCAP_0127; MCAP_0195	Serum reduced medium	Exoproteome	Voros et al., 2015
	Aminopeptidase	*M. flocculare*	ATCC 27716	WP_002557776.1	Infected cells (serum free medium)	Exoproteome	Leal Zimmer et al., 2019
		*M. hyopneumoniae*	J	AAZ44217.1	Infected cells (serum free medium)	Exoproteome	Leal Zimmer et al., 2019
	Leucyl aminopeptidase	*M. hyopneumoniae*	7448	AAZ53831.2	Infected cells (serum free medium)	Exoproteome	Leal Zimmer et al., 2019
		*M. flocculare*	ATCC 27716	WP_002557977.1	Infected cells (serum free medium)	Exoproteome	Leal Zimmer et al., 2019
		*M. bovis*	HB0801	Mbov_0789; Mbov_0673	Serum reduced medium	Exoproteome	Zubair et al., 2020b
	XAA-PRO aminopeptidase	*M. hyopneumoniae*	7448	AAZ54021.1	Infected cells (serum free medium)	Exoproteome	Leal Zimmer et al., 2019
	Peptidase M24 family protein	*M. flocculare*	ATCC 27716	WP_002557496.1	Infected cells (serum free medium)	Exoproteome	Leal Zimmer et al., 2019
	Clp protease ATP-binding subunit	*M. bovis*	HB0801	Mbov_0703	Serum reduced medium	Exoproteome	Zubair et al., 2020b

Lipases	Lipase MilA	*M. bovis*	PG45	MBOVPG45_0710	Complete growth medium	Western blotting	Adamu et al., 2020
			HB0801	ADR24994.1	Serum reduced medium	Exoproteome	Zubair et al., 2020b
	Lipase P65	*M. hyopneumoniae*	7448	AAZ54018.1	Serum reduced medium; Infected cells (serum free medium)	Exoproteome	Paes et al., 2017; Leal Zimmer et al., 2019
			J	AAZ44739.1	Infected cells (serum free medium)	Exoproteome	Leal Zimmer et al., 2019
	Triacyl glycerol lipase	*M. bovis*	HB0801	Mbov_0558	Serum reduced medium	Exoproteome	Zubair et al., 2020b

Adhesins	Protein P97	*M. hyopneumoniae*	7448	MHP7448_0198; MHP7448_0108	Serum reduced medium; Infected cells (serum free medium)	Exoproteome	Paes et al., 2017; Leal Zimmer et al., 2019
		*M. hyopneumoniae*	J	AAZ44197.1	Infected cells (serum free medium)	Exoproteome	Leal Zimmer et al., 2019
	Protein 102	*M. hyopneumoniae*	7448	MHP7448_0199; MHP7448_0107	Serum reduced medium; Infected cells (serum free medium)	Exoproteome	Paes et al., 2017; Leal Zimmer et al., 2019
		*M. hyopneumoniae*	J	AAZ44196.1; AAZ44286.1	Infected cells (serum free medium)	Exoproteome	Leal Zimmer et al., 2019
		*M. hyopneumoniae*	232	ND	Complete growth medium	Immunoelectron microscopy	Djordjevic et al., 2004; Adams et al., 2005
		*M. flocculare*	ATCC 27716	MFC_00475	Serum reduced medium	Exoproteome	Paes et al., 2017
	P216 surface protein	*M. flocculare*	ATCC 27716	MFC_00848	Serum reduced medium	Exoproteome	Paes et al., 2017
		*M. hyopneumoniae*	7448	AAZ53862.1	Infected cells (serum free medium)	Exoproteome	Leal Zimmer et al., 2019
			J	AAQ11195.1	Infected cells (serum free medium)	Exoproteome	Leal Zimmer et al., 2019
	ABC transporter xylose- binding lipoprotein	*M. hyopneumoniae*	7448	MHP7448_0604	Serum reduced medium; Infected cells (serum free medium)	Exoproteome	Paes et al., 2017; Leal Zimmer et al., 2019
		*M. flocculare*	ATCC 27716	ENX51036.1	Infected cells (serum free medium)	Exoproteome	Leal Zimmer et al., 2019
		*M. hyopneumoniae*	J	AAZ44690.2	Infected cells (serum free medium)	Exoproteome	Leal Zimmer et al., 2019
	46K surface antigen precursor	*M. hyopneumoniae*	7448	AAZ53879.1	Infected cells (serum free medium)	Exoproteome	Leal Zimmer et al., 2019
			J	P0C0J8.1	Infected cells (serum free medium)	Exoproteome	Leal Zimmer et al., 2019
		*M. flocculare*	ATCC 27716	WP_002557638.1	Infected cells (serum free medium)	Exoproteome	Leal Zimmer et al., 2019

Others proteins	Pyruvate dehydrogenase E1, beta subunit	*M. synoviae*	53	gi| 144575045	Buffer	Exoproteome	Rebollo Couto et al., 2012
		*M. hyopneumoniae*	J	AAZ44204.1	Infected cells (serum free medium)	Exoproteome	Leal Zimmer et al., 2019
	Enolase	*M. synoviae*	53	gi| 71894034	Buffer	Exoproteome	Rebollo Couto et al., 2012
		*M. capricolum* subsp. *capricolum*	Ckid	MCAP_0213	Serum reduced medium	Exoproteome	Voros et al., 2015
		*M. hyopneumoniae*	7448	AAZ53624.1	Infected cells (serum free medium)	Exoproteome	Leal Zimmer et al., 2019
			J	AAZ44333.1	Infected cells (serum free medium)	Exoproteome	Leal Zimmer et al., 2019
		*M. flocculare*	ATCC 27716	WP_002557541.1	Infected cells (serum free medium)	Exoproteome	Leal Zimmer et al., 2019
		*M. bovis*	HB0801	Mbov_0482	Serum reduced medium	Exoproteome	Zubair et al., 2020b
	Chaperone protein DnaK	*M. synoviae*	53	gi|71894366	Buffer	Exoproteome	Rebollo Couto et al., 2012
		*M. hyopneumoniae*	7448	AAZ53444.1	Infected cells (serum free medium)	Exoproteome	Leal Zimmer et al., 2019
			J	AAZ44157.1	Infected cells (serum free medium)	Exoproteome	Leal Zimmer et al., 2019
		*M. flocculare*	ATCC 27716	WP_002557920.1	Infected cells (serum free medium)	Exoproteome	Leal Zimmer et al., 2019
	Elongation factor EF-Tu	*M. synoviae*	53	gi| 71894677	Buffer	Exoproteome	Rebollo Couto et al., 2012
		*M. hyopneumoniae*	7448	AAZ53889.1	Infected cells (serum free medium)	Exoproteome	Leal Zimmer et al., 2019
			J	AAZ44610.1	Infected cells (serum free medium)	Exoproteome	Leal Zimmer et al., 2019
		*M. flocculare*	ATCC 27716	WP_002557626.1	Infected cells (serum free medium)	Exoproteome	Leal Zimmer et al., 2019
	Lipoprotein P280	*M. bovis*	HB081	AFM51648.1	Complete growth medium	Western blotting	Zhao et al., 2021
	glyceraldehyde 3-phosphate dehydrogenase	*M. hyopneumoniae*	J	AAZ44125.1	Infected cells (serum free medium)	Exoproteome	Leal Zimmer et al., 2019
			7448	AAZ53412.1	Infected cells (serum free medium)	Exoproteome	Leal Zimmer et al., 2019
		*M. flocculare*	ATCC 27716	MFC_00829	Serum reduced medium	Exoproteome	Paes et al., 2017

*Selected proteins have a known function and their extracellular localization has been demonstrated (either experimentally or because they are found in the exoproteome of several species/strains or conditions). ND, not done.*

#### Nucleases

Nucleases are easy to detect *in vitro* by visualization of DNA hydrolysis. For instance, DNA can be embedded into a solid matrix, either agar growth medium or a polyacrylamide gel, and its hydrolysis results in a clear halo around colonies or a clear band after electrophoretic migration of the nuclease (Minion et al., 1993; Sharma et al., 2015; Zhang et al., 2016; Yamamoto et al., 2017). Extracellular nucleases have been suspected in mycoplasmas since 1993, when Minion et al. demonstrated degradation of linear DNA by mycoplasma-free supernatants obtained after incubation of several mycoplasma species in a nuclease assay buffer (Minion et al., 1993). More recently, DNA degradation around *M. pneumoniae* colonies indicated the secretion of an extracellular nuclease, which was found by mutagenesis to be the MPN491 nuclease (Yamamoto et al., 2017). Other extracellular proteins with nuclease activity *in vitro* have been reported, including the P40 protein of *M. penetrans*, as demonstrated using zymography (Bendjennat et al., 1997), and the MAM superantigen of *M. arthritidis*, as demonstrated by degradation of genomic DNA (Diedershagen et al., 2007). Homologs of these nucleases have also been detected in the cytoplasmic membrane of other species, but their extracellular localization has yet not been investigated (Sharma et al., 2015).

Specific antibodies can be used to detect and localize nucleases. An example is the Mhp597 nuclease of *M. hyopneumoniae*, an ortholog of *M. pneumoniae* MPN491, which was detected in the growth medium supernatant by western blotting using specific antibodies (Li et al., 2019).

Although less straightforward, bulk proteomics data can be examined to detect potential nucleases in the cell environment. For instance, several nucleases have been identified by MS in the exoproteome purified from cell-free culture supernatants [e.g., Mhp7448_0580 (homolog of Mhp597) and MBOV_RS02825] or in the supernatant of a swine tracheal cell line infected with *M. hyopneumoniae* (Zhang et al., 2016; Paes et al., 2017; Leal Zimmer et al., 2019; Table 1).

Once secreted into the mycoplasmal environment, extracellular nucleases could participate in (i) nutrient scavenging (through degradation of the DNA released by cell lysis during the infection process) or in (ii) host evasion through hydrolysis of the neutrophil extracellular traps (NET), which are DNA networks produced by neutrophils that can trap and kill pathogens. NET evasion has been shown *in vitro* for two important lung pathogens, *M. bovis* in cattle (by nuclease MBOV_RS02825) and *M. pneumoniae* in humans (with nuclease MPN491) (Zhang et al., 2016; Yamamoto et al., 2017). The inactivation/deletion of the gene coding MPN491 resulted in a reduced survival of *M. pneumoniae* in the presence of neutrophils *in vitro* as well as *in vivo* in a mouse nasal infection model. In the most recent example, poor survival of a mutant was associated with its inability to degrade NETs induced by *Escherichia coli* lipopolysaccharides (Yamamoto et al., 2017).

#### Proteases

Extracellular proteases produced by lung-colonizing ruminant mycoplasmas have been demonstrated by measuring the degradation of fluorescent casein in culture supernatants and observing clearer digestion areas around colonies on casein-enriched solid medium (Allam et al., 2010; Ganter et al., 2019). Zymography and mutagenesis experiments further showed that *M. mycoides* subsp. *capri* MMCAP2_0241 and *M. capricolum* subsp. *capricolum* MCAP_0240, which both belong to the S41 peptidase family, are the main extracellular peptidases of these two species (Allam et al., 2010, 2012; Ganter et al., 2019). By zymography, the apparent molecular mass of these polypeptidases was estimated to be 55 kDa, in contrast with the 75 kDa molecular mass predicted from the sequences of the corresponding genes. Two predicted transmembrane domains at the N- and C-terminal ends suggest a potential membrane localization of the native proteins that may be cleaved, by endoproteolysis for example, to be released into the medium (Ganter et al., 2019).

Sequence analysis and function prediction have identified several peptidases in the exoproteome of *M. capricolum* subsp. *capricolum* (Voros et al., 2015) and *M. bovis* (Zubair et al., 2020b), as well as in the supernatant of cells infected with *M. hyopneumoniae* and *M. flocculare* (Leal Zimmer et al., 2019; Table 1).

In other bacterial models, such as *Staphylococcus* (*S.*) *aureus*, extracellular proteases are involved in nutrient acquisition, bacterial dissemination and immune evasion (Tam and Torres, 2019). In mycoplasmas, their precise roles have yet to be defined, with a few exceptions. For instance, in *M. hyopneumoniae* the endopeptidase F and the XAA-pro aminopeptidase degrade immunologically active peptides (see below), and the cell-free form of aminopeptidase AAZ44217.1 is involved in adhesion to plasminogen and heparin (Robinson et al., 2013). Extracellular peptidases of *M. capricolum* subsp. *capricolum* MCAP_0240 may have a direct or indirect role in cell surface shaving and thus modulate adhesion and immune invasion, as deletion mutants had a modified surface proteome (Ganter et al., 2019).

#### Lipases

Chromogenic substrates, such as 2-naphthyl caprylate and 2-naphthyl butyrate, have made it possible to detect lipase activity in the supernatant of *M. capricolum* subsp. *capricolum* cultures (Voros et al., 2015), but the corresponding protein has not yet been identified. Lipase activity has also been detected in two immunodominant surface proteins, the P65 lipoprotein of *M. hyopneumoniae* (Schmidt et al., 2004) and the *M. bovis* MilA protein (Wawegama et al., 2014), using assays based on hydrolysis of lipid substrates such as O-dilauryl-rac-glycero-3-glutaric acid resorufin ester, *p*-nitrophenyl caproate or *p*-nitrophenyl palmitate. Western blotting further demonstrated that one of them, the *M. bovis* MilA lipase, is released into the culture supernatant (Adamu et al., 2020) and it has also been found in the exoproteome of *M. bovis* strain HB0801 (Zubair et al., 2020b). In contrast, the P65 lipase of *M. hyopneumoniae* was not directly shown to be extracellular, but has been found in the exoproteomes of *M. hyopneumoniae* strains J and 7448 obtained from culture supernatants (Paes et al., 2017) and from the supernatant of infected cells (Leal Zimmer et al., 2019; Table 1).

In other bacteria, such as *S. aureus*, secreted extracellular lipases are involved in a broad spectrum of functions, including acquisition of lipids from the host (Delekta et al., 2018), immune evasion (Chen and Alonzo, 2019), biofilm formation, and host cell invasion (Nguyen et al., 2018). The dual potential localization—i.e., extracellular as well as cell-attached—of MilA in mycoplasmas complicates research into its function(s) (Adamu et al., 2020). MilA seems to be essential, as *M. bovis* growth is inhibited by anti-MilA antibodies, and screening of *M. bovis* transposon libraries has failed to detect a MilA mutant (Sharma et al., 2014; Josi et al., 2019; Adamu et al., 2020). Cell-free recombinant MilA has been shown to bind lipid and heparin, suggesting a putative role in the processing and transport of lipids and in adhesion to the extracellular matrix (Adamu et al., 2020).

#### Adhesins

Dozens of mycoplasma proteins have the capacity to bind to host cells or to components of the host extracellular matrix such as actin, fibronectin and glycosaminoglycans. Several of these adhesins or adhesion-related proteins, although classically described as cell surface-associated (and belonging to the surfaceome), are regularly identified in the exoproteome of several mycoplasma species (Table 1).

For instance, the P102 adhesin of *M. hyopneumoniae* has been shown, by immunoelectron microscopy after experimental infection of swine, to be localized either within mycoplasma cells or distant from them and directly attached to respiratory cilia of the experimentally infected swine (Djordjevic et al., 2004; Adams et al., 2005). P102-homologs have been identified in the exoproteomes of *M. hyopneumoniae* and *M. flocculare* (Paes et al., 2017; Leal Zimmer et al., 2019; Table 1). Other adhesins have been found in the exoproteome of *M. hyopneumoniae*, *M. flocculare* and *M. synoviae*, but no role has yet been firmly defined for them as released proteins (Table 1). A reasonable hypothesis could be that the release of adhesins from the mycoplasma cell could promote mycoplasma dispersion by limiting their adhesion to host cells and tissues, as described for other bacteria (Coutte et al., 2003). They could also be blocking anti-adhesin antibody binding to the cell, but this has yet to be demonstrated.

However, it is becoming increasingly evident that adhesins and other proteins could also play a role in degradation of the host extracellular matrix (ECM) by binding to plasminogen. This is the case for *M. hyopneumoniae* adhesin P102 (Seymour et al., 2012; Leal Zimmer et al., 2020). Binding of P102 to plasminogen resulted in increased conversion of plasminogen into plasmin, a serine protease able to degrade the ECM either directly or through activation of other enzymes, such as metalloproteases (Seymour et al., 2012). This strategy of ECM degradation by subversion of the host plasmin system is used by many other bacteria to invade and spread (Lahteenmaki et al., 2005). Non-adhesin proteins that could also contribute to degradation of the ECM include the glycolytic enzyme glyceraldehyde-3-P-dehydrogenase (GAPDH) in *M. hyorhinis* (Wang et al., 2021), the chaperone protein DNAK, GAPDH and subunit E1α of the pyruvate dehydrogenase complex (PDHB), another enzyme involved in carbon metabolism, in *M. pneumoniae* (Grundel et al., 2016; Hagemann et al., 2017), the enzyme enolase, which catalyzes the interconversion of phosphoenolpyruvate into 2-phosphoglycerate in *M. bovis* (Song et al., 2012), and the elongation factor EF-Tu, which orchestrates transport of aminoacylated tRNA to the ribosome, in *M. pneumoniae* and *M. hyopneumoniae* (Widjaja et al., 2017). These proteins have been found in the exoproteome of mycoplasmas (Table 1).

#### Disruptors of Host-Cell Metabolism

The cell-free form of the chaperone protein DnaK from *M. fermentans* was also shown to be taken up by the host cells and localize in the cytoplasm, the perinuclear space and the nucleus (Benedetti et al., 2020). Once in the cell, DnaK interacts with several host proteins, including those involved in DNA repair, like PARP1 (poly-ADP ribose polymerase-1) and USP10 (ubiquitin carboxyl-terminal hydrolase protein-10). The interaction with USP10 in turn leads to a reduction in P53 activity, which is known to have an anti-oncogenic effect (Zella et al., 2018; Benedetti et al., 2020). For bacteria, this is a way to redirect host-cell metabolism to facilitate bacterial growth (Siegl and Rudel, 2015). Using immunoprecipitation techniques with an anti-P53 monoclonal antibody, Zella et al. identified other mycoplasmal proteins able to interact with P53, one of which was the glycolytic enzyme enolase (Zella et al., 2018), which has been found in the exoproteome of five mycoplasma species (Table 1).

#### Modulators of Host Immune Response

A western blotting study recently confirmed that the MbovP280 protein, predicted *in silico* to be secreted, was effectively released by *M. bovis* cells cultured for 36 h (Zhao et al., 2021). The secreted MbovP280 protein was further shown to bind to and induce the apoptosis of bovine macrophages through a complex signaling pathway (Zhao et al., 2021). However, the effect of a recombinant protein was greater than that of whole cells expressing MbovP280. Apoptotic activity has also been described for mycoplasmal extracellular proteins with other main roles. For instance, the nucleases P40 in *M. penetrans*, Mhp597 in *M. hyopneumoniae* and RS_02825 in *M. bovis* induce apoptosis in human lymphocytes (Bendjennat et al., 1999), in swine kidney epithelial cells (Li et al., 2019), and in bovine macrophages *via* the NFkB p65 pathway (Zhang et al., 2016), respectively. Extracellular nucleases can also play a role in modulation of cytokine production. *M. hyopneumoniae* Mhp597 nuclease has been shown to suppress INFγ secretion and stimulate IL1, IL8 and TNF secretion in macrophages (Li et al., 2019). This is an important finding, as INFγ is a first-line protection against viral infection. The MAM superantigen of *M. arthritidis* has also been shown to modulate cytokine release (Mu et al., 2000). In addition to nucleases, some proteases may also have immunomodulatory effects. *M. hyopneumoniae* endopeptidase F and XAA pro-aminopeptidase, which are both found in the exoproteome (Table 1), have recently been shown to be involved in the degradation of peptides that play a role in innate immunity (Jarocki et al., 2019). These data have started to demonstrate a role of the mycoplasma releasome in evasion of the immune system and modulation of its activity, which are two important features of mycoplasma virulence (Leal Zimmer et al., 2020; Askar et al., 2021; Jiang et al., 2021; Yiwen et al., 2021).

#### Gaps and Perspectives

The combination of (i) numerous potential experimental biases (failure to pellet some mycoplasma cells, cell lysis during the purification protocol, and so on), (ii) the high proportion (circa 30%) of hypothetical proteins with no associated function found in exoproteomes, (iii) the extent of moonlighting activity in mycoplasma proteins, and (iv) underpowered *in silico* prediction capacity (Zhao et al., 2021) means that further studies complementary to studies characterizing the core exoproteome are necessary to confirm the extracellular localization of the exoproteins and decipher their role. With the growing interest in mycoplasma exoproteins, the mycoplasmology community would welcome a consensus methodology guideline to improve the quality of results and enable valuable comparisons of exoproteomes between species. For instance, specific labeling of newly synthesized proteins using new methodologies such as bioorthogonal non-canonical amino acid tagging and proximity labeling (Shin et al., 2019; Sukumaran et al., 2021) could be helpful to distinguish the exoproteome of mycoplasmas from proteins contained in a complex environment. This includes complex growth medium, the cytoplasmic compartment in case of intracellular mycoplasmas, or different host sites occupied by mycoplasmas in the course of infection [for example *M. hyopneumoniae* has been detected in the heart, kidneys, liver and spleen of pigs (Le Carrou et al., 2006; Marois et al., 2007; Woolley et al., 2012)].

Studies addressing the role of exoproteins in the interplay with the host are also essential. As the generation and use of mycoplasma mutants is still limited, approaches based on recombinant proteins are increasingly being used (Bendjennat et al., 1999; Zhang et al., 2016; Yamamoto et al., 2017; Zhao et al., 2021), but they can sometimes exaggerate the actual role of a protein because of the high concentrations tested, which do not correspond to the actual quantities secreted by the cell (Zhao et al., 2021).

The mycoplasmology community is starting to gain a global picture of the role of exoproteome interactions with host cells and with components of the host extracellular matrix and how it shapes immune evasion or modulation. However, the exoproteome composition is likely to vary over time, depending on the host context and the time since infection. Variability in exoproteome composition between species, and potentially between strains, might also explain some of the variability in virulence.

Last but not least, although some *Sec* genes have been identified *in silico* (Staats et al., 2007), further investigation is needed into the mechanisms involved in protein release by *Mycoplasma* spp. For instance, surface shaving *via* proteases could contribute to the release of exoproteins from the cell-surface, highlighting the tight connection between the surfaceome and the releasome (Raymond et al., 2013; Jarocki et al., 2015, 2019; Tacchi et al., 2016; Berry et al., 2017; Ganter et al., 2019; Machado et al., 2020). Other non-classical protein release mechanisms have been suggested such as explosive cell events or ghost cells formation (Raymond et al., 2018a). Those could contribute to free into the extracellular medium membrane or cytoplasmic proteins (Wang et al., 2013).

### Exopolysaccharides

#### Composition and Structure

Mycoplasma exopolysaccharides were first reported in the 1930s, when Kurotchkin described a carbohydrate released by *M. mycoides* subsp. *mycoides* into the culture medium and the blood of animals with acute CBPP (Kurotchkin, 1937; Kurotchkin and Benaradsky, 1938). It was then of unknown composition and structure, but cross-reacted serologically with the *M. mycoides* subsp. *mycoides* capsular polysaccharide (Plackett et al., 1963), which is composed of galactose and named galactan (Plackett and Buttery, 1958). By the 1960s, it was clear that *M. mycoides* subsp. *mycoides* produced both a CPS and an EPS, with a shared antigenic signature recognized by the same antibodies. Despite several efforts, purification of the EPS from culture supernatants remained thwarted by difficulties in eliminating contamination from polysaccharides, such as glycogen, contained in the growth medium (Plackett et al., 1963; Hudson et al., 1967). It was only in 2013 that Bertin et al. eliminated contamination with medium-associated polysaccharides by transferring PPLO-grown *M. mycoides* subsp. *mycoides* cells into CMRL, a defined cell culture medium with no polysaccharides and only glucose as a carbon source (Bertin et al., 2013). CMRL was shown to sustain mycoplasmal metabolism but not growth (Bertin et al., 2013). An EPS, in free form in the spent CMRL, was purified and its composition and structure were shown by NMR and HPLC to be identical to the polysaccharide moiety of the capsular galactan described 50 years earlier (Bertin et al., 2013). This identity explained the immunological cross-reactivity between the EPS and CPS, but was limited to the polysaccharide moiety, as galactan CPS contains a lipid anchor (Buttery and Plackett, 1960) that is not present when the galactan is released from the cells (Bertin et al., 2013).

Other EPS were identified among the members of the *M. mycoides* cluster using the same purification method: a β-(1→2)-glucopyranose was detected in the culture supernatant of *M. capricolum* subsp. *capricolum*, *M. capricolum* subsp. *capripneumoniae* and *M. leachii* (Bertin et al., 2015; Table 2), and weak exopolysaccharide release was detected *in vitro* from the two serovars of *M. mycoides* subsp. *capri* (Bertin et al., 2015; Gaurivaud et al., 2016). Interestingly, the two serovars of the goat pathogen *M. mycoides* subsp. *capri* secreted two different polysaccharides: serovar LC (large colony) produced galactan and serovar capri produced β-(1→6)-glucopyranose. Both EPS were homopolysaccharides with no ramification and no chemical modifications. However, not all mycoplasmal polysaccharides are as simple. The EPS isolated from *M. pneumoniae* biofilms, for instance, is composed of galactose and N-acetyl glucosamine (Simmons et al., 2013; Table 2).

**TABLE 2:** List of *Mycoplasma* (sub)species producing exopolysaccharides.

Species	Hosts	Strains	Culture conditions	Tools	Polysaccharides		Biosynthesis pathways	CPS/EPS	References
					Composition	Structures			
*M. pneumoniae*	Human	M129	Biofilm	GC	Galactose, GlcNac	ND	ND	EPS	Simmons et al., 2013
		UAB PO1	Biofilm	GC	Galactose, GlcNac	ND	ND	CPS	Simmons et al., 2013

*M. mycoides* subsp. *mycoides*	Bovine	V5	Cell grown in complex medium	Biochemical and optical methods	Galactose	β-(1→6)-galactofuranose	Synthase	CPS	Plackett and Buttery, 1958, 1964
		Afadé	Cell incubated in CMRL-medium	HPLC, NMR, MAb	Galactose	β-(1→6)-galactofuranose	Synthase	CPS/EPS	Bertin et al., 2013, 2015
*M. mycoides* subsp. *capri* serovar capri	Caprine	PG3^T^	Cell grown in complex medium	HPLC, NMR	Glucose	β-(1→6)-glucopyranose	Synthase	CPS/EPS	Gaurivaud et al., 2016
*M. mycoides* subsp. *capri* serovar LC	Caprine	95010	Cell grown in complex medium	MAb	Galactose	β-(1→6)-galactofuranose	Synthase	CPS/EPS	Bertin et al., 2015

*M. capricolum* subsp. *capricolum*	Caprine	7714	Cell grown in complex medium	MAb	Glucose	β-(1→2)-glucopyranose	Synthase	CPS/EPS	Bertin et al., 2015
*M. capricolum* subsp. *capripneumoniae*	Caprine	Ambosa	Cell grown in complex medium	MAb	Glucose	β-(1→2)-glucopyranose	Synthase	CPS/EPS	Bertin et al., 2015

*M. leachii*	Bovine	PG50^T^	Cell incubated in CMRL-medium	HPLC, NMR	Glucose	β-(1→2)-glucopyranose	Synthase	EPS	Bertin et al., 2015
			Cell grown in complex medium	MAb	Glucose	β-(1→2)-glucopyranose	Synthase	CPS	Bertin et al., 2015

*M. agalactiae*	Caprine	14628	Cell grown in complex medium	HPLC, NMR, Mab	Glucose	β-(1→6)-glucopyranose	Synthase	CPS	Gaurivaud et al., 2016

*M. feriruminatoris*	Ibex	15568	Cell grown in complex medium	HPLC, NMR, MAb	Glucose, galactose	β-(1→6)-glucopyranose, β-(1→6)-galactofuranose	Synthase	CPS	Ambroset et al., 2017

*ND, not determined; Mab, monoclonal antibody; HPLC, high pressure liquid chromatography; NMR, nuclear magnetic resonance; CPS, cell-linked polysaccharide; EPS, exopolysaccharide; GlcNac, N-acetylglucosamine; PNAG, Poly-N-acetylglucosamine; T, type strain; GC, gas chromatography.*

All these EPS were also detected as CPS (Table 2). However, the presence of a CPS does not guarantee secretion of the corresponding EPS, as shown by the β-(1→6)-glucopyranose of *M. agalactiae*, which was only detected as a CPS (Gaurivaud et al., 2016; Table 2). Other CPS have been detected in *M. feriruminatoris*, which is able to produce a galactan and a β-(1→6)-glucopyranose CPS (Ambroset et al., 2017), and in *M. genitalium* (Daubenspeck et al., 2020) and *M. pulmonis* (Daubenspeck et al., 2009; Table 2). However, their release as EPS has not been demonstrated to date.

#### Exopolysaccharides Biosynthesis

In *M. mycoides* subsp. *mycoides*, the galactan is not secreted concomitantly as CPS and EPS *in vitro*, but rather alternatively by phenotypic variants undergoing phase variation (Bertin et al., 2013). One variant secreted a galactan CPS but no EPS, whereas the other one was not capsulated but produced a galactan EPS. This phenomenon was reversible and was shown to be related to the expression of a permease of the glucose-phosphoenolpyruvate phosphotransferase system (PTS) undergoing on/off phase variation (Gaurivaud et al., 2004; Bertin et al., 2013). The PTS system is a well-established sugar transport system, but also regulates carbon metabolism in bacteria (Deutscher et al., 2014) and has already been shown to regulate polysaccharide synthesis in *Vibrio cholerae* (Houot et al., 2010). One group posited glucose PTS system-mediated regulation of a glycosyltransferase possibly involved in attachment of the polysaccharide moiety to the membrane lipid anchor (Bertin et al., 2013), but the regulatory and attachment mechanisms have yet to be demonstrated.

A complete biosynthesis pathway involving a membrane glycosyltransferase belonging to the synthase family (Whitney and Howell, 2013), and catalyzing both polymerization and transfer of the galactan of *M. mycoides* subsp. *mycoides* in its CPS or EPS across the cytoplasmic membrane, has been proposed based on *in silico* data (Bertin et al., 2013). The role of synthases in mycoplasma polysaccharide synthesis was also demonstrated using a functional genomics approach for the synthesis of the *M. agalactiae* β-(1→6)-glucopyranose (Gaurivaud et al., 2016). Among the many glycosyltransferases identified in mycoplasmal genomes, synthases are easily identified as they have 4 or 7 transmembrane domains and a cytoplasmic loop bearing the glycosyltransferase-active sites (Gaurivaud et al., 2016). The number of transmembrane domains may be linked to substrate specificity and the structure of the resulting polymer. Synthases have been predicted *in silico* for 14 *Mycoplasma* species, which suggests that several other polysaccharides have yet to be identified (Gaurivaud et al., 2016). In contrast, no synthesis pathway has been identified for the polysaccharides produced by *M. pulmonis*, *M. genitalium* or *M. pneumoniae* (Daubenspeck et al., 2009, 2020). However, an ABC transporter pathway (Schmid, 2018) has been suggested for *M. pulmonis*, as the mutation of two ABC permease genes was associated with a loss of polysaccharide production (Daubenspeck et al., 2009).

#### Role in Host Interactions

In mycoplasmas, as in other bacterial models, CPS are known to be involved in the modulation of cytoadherence (Bolland and Dybvig, 2012) and in protection against phagocytosis (Shaw et al., 2013) and against the bactericidal activity of the complement system (Bolland et al., 2012; Gaurivaud et al., 2014). In contrast, with the exception of circulating galactan in the body fluids of animals experiencing CBPP, the role of mycoplasmal EPS has been under-researched. Intravenous injection of galactan purified from *M. mycoides* subsp. *mycoides* culture supernatant into calves resulted in an increase of pulmonary arterial blood pressure and a transient apnea (Buttery et al., 1976). However, in its EPS form, the galactan did not induce any fever and did not affect the susceptibility of calves to subsequent subcutaneous infection with *M. mycoides* subsp. *mycoides* (Hudson et al., 1967; Buttery et al., 1976). However, these results need to be interpreted with caution, as the purified galactan was shown to contain peptides (Hudson et al., 1967; Buttery et al., 1976). This ambiguity was overcome recently by Totte et al. (2015) who used a high-purity-grade galactan obtained from *M. mycoides* subsp. *mycoides* supernatant, checked by SDS PAGE and NMR, to examine its effect on immune cells. Pure galactan failed to activate naive lymphocytes but induced IL-10 release by bovine macrophages, thus echoing the observation of peak IL-10 1–2 weeks after experimental infection of cattle with *M. mycoides* subsp. *mycoides* (Sacchini et al., 2012). Moreover, the purified free galactan was able to reduce the release of pro-inflammatory cytokines by macrophages in response to *Escherichia coli* lipopolysaccharide (Totte et al., 2015). These observations indicated that, overall, the galactan EPS acts as an immunosuppressor by inhibiting pro-inflammatory cytokines and increasing the IL-10 secretion that depresses T-cell responses (Totte et al., 2015).

Polysaccharides are a major constituent of biofilm matrices, where they play roles in binding different components and in physical resistance to stress or stratification of the biofilm structure (Limoli et al., 2015). Both CPS and EPS could contribute to biofilm formation in mycoplasmas. For instance, in *M. pulmonis*, different types of expressed dominant polysaccharides changed the propensity of strains to form a biofilm (Daubenspeck et al., 2009). Similarly, in *M. pneumoniae*, the volume, texture, robustness and internal structure of the biofilm was shown to differ between two strains (of different type), depending on how loosely or tightly the polysaccharides were attached to the mycoplasma cell (Simmons et al., 2013). This could ultimately completely modify the role of the resulting biofilm in virulence and chronicity of infection.

#### Gaps and Perspectives

Although extracellular polysaccharides have been detected in several *Mycoplasma* species, only three polymers have had their structure elucidated thus far. This relatively low rate of structural characterization may be attributable to the necessity of purifying a high quantity (approximately 1 mg) of material for structure analyses by HPLC and NMR, which could be technically challenging depending on the species, culture conditions and overall complexity of the exopolysaccharide structure (e.g., biofilms) (Table 2).

Clear identification of the pathways involved in the synthesis of galactan, β-(1→6)-glucopyranose and β-(1→2)-glucopyranose has been helpful for *in silico* screening of other species for their potential to produce polysaccharides. However, for *M. pulmonis*, *M. genitalium* and *M. pneumoniae*, the EPS biosynthesis pathways have yet to be deciphered. In *M. pulmonis*, random transposon mutagenesis identified the role of two genes coding for ABC transporters, but failed to find the glycosyltransferase catalyzing the polymerization. This suggests that polymerization of the polysaccharides could be an essential function for mycoplasmas. Other mutations in genes involved in polysaccharide biosynthesis were shown to have an adverse effect on cell viability: for example, a mutation in the UDP galactofuranose mutase gene of *M. mycoides* subsp. *capri* reduced the membrane integrity of the cells (Schieck et al., 2016). The genes involved in polysaccharide attachment to the cell surface and the membrane anchor have not yet been identified.

A final crucial point is that we are still a long way from identifying the roles of EPS and their level of secretion *in vivo*. The example of galactan illustrates that, despite having the same polysaccharide moiety, CPS and EPS have two different roles: protection of the cell (CPS) and suppression of inflammation (EPS). This has some similarity with the moonlighting proteins of mycoplasmas that play different roles depending on their localization. Phase variation between capsulated variants and uncapsulated variants secreting EPS is thought to be involved in the adaptation of *M. mycoides* subsp. *mycoides* to changing environments during host colonization (Gaurivaud et al., 2014), but this hypothesis has yet to be validated *in vivo*.

### Extracellular Vesicles

#### First Observations

Extracellular vesicles are the least investigated elements of the mycoplasmal releasome. Membranous particles, with a diameter of 75 to 210 nm, were first observed in the 1960s by electron microscopy in mycoplasma cultures during studies of cell ultrastructure and cellular division (Domermuth et al., 1964; Hummeler et al., 1965). More recently, using a classical method for EV purification, similar nanosized particles were observed by TEM in *Acholeplasma laidlawii* and several *Mycoplasma* species (Chernov et al., 2011; Gaurivaud et al., 2018). The diameters of these particles ranged from 30 to 170 nm, although a few larger particles of around 200 nm were also observed for *M. mycoides* subsp. *mycoides* (Gaurivaud et al., 2018). Although the shapes and sizes of these particles were typical of classical prokaryotic EV, further evidence was needed to definitively confirm that they were EV: they had to be (i) non-replicative, (ii) surrounded by a lipid bilayer, (iii) produced by viable cells, and (iv) contain cytoplasmic proteins in order to prove that the particle was not just a circularization/reassembly of membrane fragments (Thery et al., 2018). They were shown to meet the first three criteria (Gaurivaud et al., 2018), but the fourth criterion—internal protein contents of the EV—has not yet been assessed. However, TEM images of mycoplasma cultures clearly showed EV budding from cells, suggesting that they are not the result of reassembling membrane fragments nor of an aberrant division of small mycoplasma cells (Hummeler et al., 1965; Gaurivaud et al., 2018).

Production of vesicles by exploding bacterial cells has been described recently for *Bacillus* spp. (Toyofuku et al., 2019). The “explosion” results from the expression of an autolysin, i.e., a peptidoglycan hydrolyzing enzyme. After the degradation of the cell-wall, the cell is lysed because of the high intracellular pressure [10 atm for *Bacillus* (Rojas and Huang, 2018)]. Subsequent reassembly of bacterial membrane debris can generate vesicles. However, these vesicles differ from surface-budding EV with respect to their composition (Toyofuku et al., 2019). Explosive cell-lysis events leading to membrane vesicles formation were once observed for *M. hyopneumoniae* cells embedded in a biofilm produced *in vitro* (Raymond et al., 2018a). Comparison of size, structure and cargo composition (expected to be random in case of explosive cells) between EV and vesicles produced from explosive cell events would certainly shed new light onto their respective roles.

Bacterial EV are involved in an array of processes, including stress responses, cell communication, and protein secretion (Kim et al., 2015; Coelho and Casadevall, 2019). Several proteins involved in host interactions have been identified in the proteome of EV membranes in three species, including pathogens of both domestic animals (*M. mycoides* subsp. *mycoides* and *M. agalactiae*) and humans (*M. fermentans*) (Gaurivaud et al., 2018). Of all the putative virulence factors found in EV membranes, DnaK is one of the most abundant proteins (Gaurivaud et al., 2018) and this protein has also been detected in host cells (Zella et al., 2018) (see earlier). Secretion through EV and delivery into the host cells could be used by mycoplasmas that are devoid of other more classical secretion systems. Secretion of proteins through EV confers both protection of the cargo molecules against degradation and an efficient transport system able to deliver high concentrations of cargo molecules to the cells (Coelho and Casadevall, 2019; McMillan and Kuehn, 2021).

Bacterial EV could be involved in nutrient acquisition, as recently described for iron acquisition by EV of *Mycobacterium tuberculosis* and *Pseudomonas* (*P.*) *aeruginosa* (Prados-Rosales et al., 2014; Lin et al., 2017). These bacteria are able to scavenge the iron sequestered by EV, and because of their dissemination over long distances, EV are assumed to supply nutrients to bacteria localized at different infection sites. Given that nutrient binding proteins, such as those belonging to ABC transporter systems, have been identified in the EV membranes of mycoplasmas (Gaurivaud et al., 2018), EV could participate in nutrient acquisition, which is a crucial feature for these biosynthetically limited bacteria.

#### Gaps and Perspectives

Data on the biogenesis, composition and role of mycoplasmal EV are scarce. Despite the considerable efforts made to date, there is still a need to further optimize the purification of EV (Thery et al., 2018) and completely eliminate the small-sized mycoplasma cells observed in the purified material from some species (Gaurivaud et al., 2018). A second gap to address is to complete the characterization of the EV composition and, crucially, their cytoplasmic content. The presence of polypeptides, nucleic acids, DNA and RNA within EV has not been yet assessed. A method that can directly assess the presence of DNA or RNA inside EV has been proposed recently (Bitto et al., 2017).

Extracellular vesicles secretion is energetically expensive for bacteria (McMillan and Kuehn, 2021) and may be even more expensive for the small-sized (300–800 nm diameter) mycoplasmas (Razin et al., 1998), which produce EV of 30–170 nm diameter. This raises the question of the fitness burden that EV release might represent for mycoplasmas, which needs to be compensated for by beneficial roles of EV release. Remodeling mycoplasmas membrane by vesicle production could be a rapid mechanism to maintain membrane integrity and its adaptation to changing environments, as it is described for other bacteria (McMillan and Kuehn, 2021). Deciphering the cargo composition of EV, and an in-depth comparison of virulence factors in the vesicle membranes (Gaurivaud et al., 2018) with those of parental cells, would certainly help define these beneficial roles. Selective cargo packaging, i.e., preferential exclusion or selection of vesicle cargo, has already been demonstrated in enterotoxigenic *Escherichia coli* during stress (Orench-Rivera and Kuehn, 2021). EV produced by explosive-cell events [observed for *M. hyopneumoniae* biofilm *in vitro* (Raymond et al., 2018a)] carry different cargo than classical EV and may have different roles (Toyofuku et al., 2019; McMillan and Kuehn, 2021) such as release of “public goods” useful for the bacterial community inside the biofilm (Turnbull et al., 2016).

The *in vitro* use of cellular models to study EV release and cytotoxicity or their effect on immunity compared to mycoplasmas alone would bring further insights into their role. Similarly, experimental infection of animals could provide information about the potential long-distance dissemination of mycoplasmal EV (Stentz et al., 2018).

Extracellular vesicles-based vaccines could also hold promise for use in mycoplasmosis control as it has been shown for other bacterial diseases (Jiang et al., 2019; Behrens et al., 2021). However, the presence of proinflammatory lipoproteins in the mycoplasmal EV membrane and the yield rate and cost of *in vitro* EV production remain technical and economic bottlenecks, although they could be solved by expressing extracellular virulence factors within heterologous EV systems (Carvalho et al., 2019; Stentz et al., 2022).

### Other Components of the Releasome

In addition to the three main elements of the releasome (exoproteins, EPS, and EV), mycoplasmas can also release other molecules, including DNA and byproducts of mycoplasmal metabolism. Further insights into the biology of mycoplasmas came from the recent demonstration within *M. hyopneumoniae* of morphological variants called ‘large cell variants’ (LCV) with inherent membrane instability and hence a capacity to release their cytoplasmic content, notably extracellular DNA by cell lysis leading to the formation of ghost cells and to explosive cell events (Raymond et al., 2018a). As extracellular DNA is necessary for biofilm formation on abiotic surfaces, Raymond et al. proposed that mycoplasmas could be self-sufficient in providing this DNA through LCV lysis. The contribution of LCV cell lysis to the final composition of the releasome has yet to be further defined.

Two byproducts of mycoplasmal metabolism, hydrogen peroxide and hydrogen sulfide, have drawn attention because of their potential cytotoxicity. Hydrogen sulfide is produced during cysteine catabolism by cysteine desulfurase/desulfhydrase encoded by the *Hap*E gene, which is found in many mycoplasmas (Grosshennig et al., 2016). Hydrogen peroxide is produced through glycerol metabolism by L-α-glycerophosphate oxidase or peroxide hydrogen NADH oxidase, and many mycoplasmas are able to produce it (Khan et al., 2005; Blotz and Stulke, 2017; Zhao et al., 2017). These two metabolites are considered virulence factors because of their capacity to damage their host cells (Blotz and Stulke, 2017). Hydrogen sulfide produced by *M. pneumoniae* has hemolytic activity (Grosshennig et al., 2016). Hydrogen peroxide, a known virulence factor of *Streptococcus pneumoniae* (Gonzales et al., 2021), may be a virulence factor in several *Mycoplasma* species (Pilo et al., 2005; Hames et al., 2009; Zhu et al., 2019). In addition, hydrogen peroxide could prevent growth of competing bacteria (Herrero et al., 2016), thus offering an advantage in niche colonization.

## Conclusion

Mycoplasmas actively release a wide variety of molecules and elements—from metabolites to proteins, polysaccharides, DNA and EV—into their environment. Not all these elements are associated with a known secretion system, but their common extracellular localization provides grounds for gathering them together under the general term of *releasome*. Besides molecules and elements actively released by living cells, cell lysis leading to formation of ghosts cells and explosive cells event could be a mechanism for generating a certain releasome in a biofilm context (Raymond et al., 2018a).

Characterization of the releasome of *Mycoplasma* spp. began with the first culture of a mycoplasma, in 1898. To parallel with other bacteria, the first extracellular elements searched for were toxins and other virulence factors. Despite the methodological bottlenecks of the time, proteases, lipases and nucleases in the extracellular environment were detected early on, but the corresponding secretion machineries and their substrates were not identified. With time, another difficulty has arisen, as most of the released elements or parts of them (for instance, the polysaccharide moiety of cell-linked galactan or the external region of membrane proteases) also have a cell-localized counterpart that could have a different function, highlighting the tight connection between the releasome and the surfaceome. EVs have an important role in the releasome as they can contain proteins, polysaccharides and DNA in a protected environment. The physical form (free vs. EV-associated) of various elements of the releasome may modify their role, as has been described for LPS-induced activation of the inflammasome in *P. aeruginosa* (Bitto et al., 2018).

Data collected for the purpose of this review show that the elements of the releasome are now recognized as playing important roles in nutrient acquisition, adhesion to and invasion of host cells, and immune system modulation and evasion. However, a better understanding of the global role and dynamics of the releasome hinges on studying the balance of its different components (proteins, polysaccharides, metabolites and EV) at a precise time point after infection of the host, in different host sites, in specific physiological states of mycoplasmas, including their intracellularity.

Further advances in deciphering the releasome of mycoplasmas will bring further knowledge and hypotheses about the interplay between mycoplasmas and their hosts and help to progress identification of new molecules involved in virulence or cytoplasmic or membrane molecules that have different roles once they are released from the cell. Such virulence factors are not included in current inactivated vaccines. Moreover, exoproteome modifications have been observed after *in vitro* serial passage (Zhang et al., 2021), which indicates that the releasome of passage-attenuated vaccine strains may differ from that of virulent field strains. Ultimately, experiments are needed to assess the benefit of adding releasome components to current vaccines.

## Definitions

*Releasome:* set of not cell-attached molecules (proteins, polysaccharides, DNA and metabolites) and elements (EV) released by a living cell into its extracellular environment, whatever the release/secretion mechanism, but excluding release by cell lysis and contamination by medium components. As opposed to the secretome, the machinery of secretion may be unknown.

*Secretome:* classically defined as the set of proteins and their corresponding secretion systems allowing translocation through the membrane from the inside to the outside of the cell. This implies that the secretion systems are known.

*Exoprotein*: a protein released by viable mycoplasmas into their environment. This excludes cell-attached proteins (surfaceome).

*Exoproteome*: set of exoproteins secreted by a cell in a defined time and environment.

*Extracellular vesicle (EV)*: nanosized, membranous spherical structure produced from viable cells by budding. EVs are composed of a part of the membrane and cytoplasm of the parental cell, but cargo selection means that their composition is not the same as the parental cell.

*Cell-linked polysaccharide (CPS):* polysaccharide covalently linked to the cell surface. The resulting polymer remains attached to the cell where it can form a capsule or a slime layer around the cells. CPS are purified from washed cells.

*Exopolysaccharide (EPS):* polysaccharide non-covalently linked to the cell surface and released free in the culture supernatant or within the host. Such polysaccharides are purified *in vitro* from a cell-free supernatant.

## Author Contributions

PG and FT conceptualized, wrote the first manuscript draft, and revised the manuscript. All authors have read and approved the final version of the manuscript.

## Conflict of Interest

The authors declare that the research was conducted in the absence of any commercial or financial relationships that could be construed as a potential conflict of interest. The reviewer GB is currently organizing a Research Topic with the author FT.

## Publisher’s Note

All claims expressed in this article are solely those of the authors and do not necessarily represent those of their affiliated organizations, or those of the publisher, the editors and the reviewers. Any product that may be evaluated in this article, or claim that may be made by its manufacturer, is not guaranteed or endorsed by the publisher.

## References

[B1] Adams C. Pitzer J. Minion F. C. (2005). *In vivo* expression analysis of the P97 and P102 paralog families of *Mycoplasma hyopneumoniae*. *Infect. Immun.* 73 7784–7787. 10.1128/IAI.73.11.7784-7787.2005 16239586 PMC1273896

[B2] Adamu J. Y. Wawegama N. K. Kanci Condello A. Marenda M. S. Markham P. F. Browning G. F. (2020). *Mycoplasma bovis* membrane protein MilA is a multifunctional lipase with novel lipid and glycosaminoglycan binding activity. *Infect. Immun.* 88:e00945-19. 10.1128/IAI.00945-19 32253247 PMC7240078

[B3] Afshar A. (1967). The growth of *Mycoplasma bovigenitalium* in cell cultures. *J. Gen. Microbiol.* 47 103–110. 10.1099/00221287-47-1-103 4291869

[B4] Allam A. B. Brown M. B. Reyes L. (2012). Disruption of the S41 peptidase gene in *Mycoplasma mycoides capri* impacts proteome profile, H(2)O(2) production, and sensitivity to heat shock. *PLoS One* 7:e51345. 10.1371/journal.pone.0051345 23300541 PMC3534093

[B5] Allam A. B. Reyes L. Assad-Garcia N. Glass J. I. Brown M. B. (2010). Enhancement of targeted homologous recombination in *Mycoplasma mycoides* subsp. *capri* by inclusion of heterologous recA. *Appl. Environ. Microbiol.* 76 6951–6954. 10.1128/AEM.00056-10 20802067 PMC2953016

[B6] Ambroset C. Pau-Roblot C. Game Y. Gaurivaud P. Tardy F. (2017). Identification and characterization of *Mycoplasma feriruminatoris* sp. *nov*. strains isolated from Alpine ibex: a 4th species in the *Mycoplasma mycoides* cluster hosted by non-domesticated ruminants? *Front. Microbiol.* 8:939. 10.3389/fmicb.2017.00939 28611743 PMC5447728

[B7] Armengaud J. Christie-Oleza J. A. Clair G. Malard V. Duport C. (2012). Exoproteomics: exploring the world around biological systems. *Expert Rev. Proteomics* 9 561–575. 10.1586/epr.12.52 23194272

[B8] Askar H. Chen S. Hao H. Yan X. Ma L. Liu Y. (2021). Immune evasion of *Mycoplasma bovis*. *Pathogens* 10:297. 10.3390/pathogens10030297 33806506 PMC7998117

[B9] Atkin C. L. Wei S. Cole B. C. (1994). The *Mycoplasma arthritidis* superantigen MAM: purification and identification of an active peptide. *Infect. Immun.* 62 5367–5375. 10.1128/iai.62.12.5367-5375.1994 7960116 PMC303277

[B10] Behrens F. Funk-Hilsdorf T. C. Kuebler W. M. Simmons S. (2021). Bacterial membrane vesicles in pneumonia: from mediators of virulence to innovative vaccine candidates. *Int. J. Mol. Sci.* 22:3858. 10.3390/ijms22083858 33917862 PMC8068278

[B11] Bendjennat M. Blanchard A. Loutfi M. Montagnier L. Bahraoui E. (1997). Purification and characterization of *Mycoplasma penetrans* Ca2+/Mg2+-dependent endonuclease. *J. Bacteriol.* 179 2210–2220. 10.1128/jb.179.7.2210-2220.1997 9079906 PMC178957

[B12] Bendjennat M. Blanchard A. Loutfi M. Montagnier L. Bahraoui E. (1999). Role of *Mycoplasma penetrans* endonuclease P40 as a potential pathogenic determinant. *Infect. Immun.* 67 4456–4462. 10.1128/IAI.67.9.4456-4462.1999 10456886 PMC96764

[B13] Benedetti F. Cocchi F. Latinovic O. S. Curreli S. Krishnan S. Munawwar A. (2020). Role of *Mycoplasma* chaperone DnaK in cellular transformation. *Int. J. Mol. Sci.* 21:1311. 10.3390/ijms21041311 32075244 PMC7072988

[B14] Berry I. J. Jarocki V. M. Tacchi J. L. Raymond B. B. A. Widjaja M. Padula M. P. (2017). N-terminomics identifies widespread endoproteolysis and novel methionine excision in a genome-reduced bacterial pathogen. *Sci. Rep.* 7:11063. 10.1038/s41598-017-11296-9 28894154 PMC5593965

[B15] Bertin C. Pau-Roblot C. Courtois J. Manso-Silvan L. Tardy F. Poumarat F. (2015). Highly dynamic genomic loci drive the synthesis of two types of capsular or secreted polysaccharides within the *Mycoplasma mycoides* cluster. *Appl. Environ. Microbiol.* 81 676–687. 10.1128/AEM.02892-14 25398856 PMC4277593

[B16] Bertin C. Pau-Roblot C. Courtois J. Manso-Silvan L. Thiaucourt F. Tardy F. (2013). Characterization of free exopolysaccharides secreted by *Mycoplasma mycoides* subsp. *mycoides*. *PLoS One* 8:e68373. 10.1371/journal.pone.0068373 23869216 PMC3711806

[B17] Bitto N. J. Baker P. J. Dowling J. K. Wray-McCann G. De Paoli A. Tran L. S. (2018). Membrane vesicles from *Pseudomonas aeruginosa* activate the noncanonical inflammasome through caspase-5 in human monocytes. *Immunol. Cell Biol.* 96 1120–1130. 10.1111/imcb.12190 30003588

[B18] Bitto N. J. Chapman R. Pidot S. Costin A. Lo C. Choi J. (2017). Bacterial membrane vesicles transport their DNA cargo into host cells. *Sci. Rep.* 7:7072. 10.1038/s41598-017-07288-4 28765539 PMC5539193

[B19] Blotz C. Stulke J. (2017). Glycerol metabolism and its implication in virulence in *Mycoplasma*. *FEMS Microbiol. Rev.* 41 640–652. 10.1093/femsre/fux033 28961963

[B20] Bolland J. R. Dybvig K. (2012). *Mycoplasma pulmonis* Vsa proteins and polysaccharide modulate adherence to pulmonary epithelial cells. *FEMS Microbiol. Lett.* 331 25–30. 10.1111/j.1574-6968.2012.02551.x 22428866 PMC3343211

[B21] Bolland J. R. Simmons W. L. Daubenspeck J. M. Dybvig K. (2012). Mycoplasma polysaccharide protects against complement. *Microbiology (Reading)* 158(Pt. 7) 1867–1873. 10.1099/mic.0.058222-0 22504437 PMC3542145

[B22] Bove J. M. (1999). The one-hundredth anniversary of the first culture of a mollicute, the contagious bovine peripneumonia microbe, by Nocard and Roux, with the collaboration of Borrel, Salimbeni, and Dujardin-Baumetz. *Res. Microbiol.* 150 239–245. 10.1016/s0923-2508(99)80048-5 10376485

[B23] Bryan B. A. Linhardt R. J. Daniels L. (1986). Variation in composition and yield of exopolysaccharides produced by *Klebsiella* sp. strain K32 and *Acinetobacter calcoaceticus* BD4. *Appl. Environ. Microbiol.* 51 1304–1308. 10.1128/aem.51.6.1304-1308.1986 3729401 PMC239062

[B24] Burki S. Gaschen V. Stoffel M. H. Stojiljkovic A. Frey J. Kuehni-Boghenbor K. (2015). Invasion and persistence of *Mycoplasma bovis* in embryonic calf turbinate cells. *Vet. Res.* 46:53. 10.1186/s13567-015-0194-z 25976415 PMC4432498

[B25] Buttery S. H. Plackett P. (1960). A specific polysaccharide from *Mycoplasma mycoides*. *J. Gen. Microbiol.* 23 357–368. 10.1099/00221287-23-2-357 13689482

[B26] Buttery S. H. Lloyd L. C. Titchen D. A. (1976). Acute respiratory, circulatory and pathological changes in the calf after intravenous injections of the galactan from *Mycoplasma mycoides* subsp. *mycoides*. *J. Med. Microbiol.* 9 379–391. 10.1099/00222615-9-4-379 794475

[B27] Caradec J. Kharmate G. Hosseini-Beheshti E. Adomat H. Gleave M. Guns E. (2014). Reproducibility and efficiency of serum-derived exosome extraction methods. *Clin. Biochem.* 47 1286–1292. 10.1016/j.clinbiochem.2014.06.011 24956264

[B28] Carvalho A. L. Miquel-Clopes A. Wegmann U. Jones E. Stentz R. Telatin A. (2019). Use of bioengineered human commensal gut bacteria-derived microvesicles for mucosal plague vaccine delivery and immunization. *Clin. Exp. Immunol.* 196 287–304. 10.1111/cei.13301 30985006 PMC6514708

[B29] Cerning J. (1990). Exocellular polysaccharides produced by lactic acid bacteria. *FEMS Microbiol. Rev.* 7 113–130. 10.1111/j.1574-6968.1990.tb04883.x 1702979

[B30] Chagnot C. Zorgani M. A. Astruc T. Desvaux M. (2013). Proteinaceous determinants of surface colonization in bacteria: bacterial adhesion and biofilm formation from a protein secretion perspective. *Front. Microbiol.* 4:303. 10.3389/fmicb.2013.00303 24133488 PMC3796261

[B31] Chen X. Alonzo F. III (2019). Bacterial lipolysis of immune-activating ligands promotes evasion of innate defenses. *Proc. Natl. Acad. Sci. U.S.A.* 116 3764–3773. 10.1073/pnas.1817248116 30755523 PMC6397559

[B32] Chernov V. M. Chernova O. A. Mouzykantov A. A. Efimova I. R. Shaymardanova G. F. Medvedeva E. S. (2011). Extracellular vesicles derived from *Acholeplasma laidlawii* PG8. *ScientificWorldJournal* 11 1120–1130. 10.1100/tsw.2011.109 21623458 PMC5719990

[B33] Chernov V. M. Mouzykantov A. A. Baranova N. B. Medvedeva E. S. Grygorieva T. Y. Trushin M. V. (2014). Extracellular membrane vesicles secreted by mycoplasma *Acholeplasma laidlawii* PG8 are enriched in virulence proteins. *J. Proteomics* 110 117–128. 10.1016/j.jprot.2014.07.020 25088052

[B34] Coelho C. Casadevall A. (2019). Answers to naysayers regarding microbial extracellular vesicles. *Biochem. Soc. Trans.* 47 1005–1012. 10.1042/BST20180252 31320501 PMC11386541

[B35] Coutte L. Alonso S. Reveneau N. Willery E. Quatannens B. Locht C. (2003). Role of adhesin release for mucosal colonization by a bacterial pathogen. *J. Exp. Med.* 197 735–742. 10.1084/jem.20021153 12629063 PMC2193847

[B36] Daubenspeck J. M. Bolland J. R. Luo W. Simmons W. L. Dybvig K. (2009). Identification of exopolysaccharide-deficient mutants of *Mycoplasma pulmonis*. *Mol. Microbiol.* 72 1235–1245. 10.1111/j.1365-2958.2009.06720.x 19432800 PMC2752295

[B37] Daubenspeck J. M. Totten A. H. Needham J. Feng M. Balish M. F. Atkinson T. P. (2020). *Mycoplasma genitalium* biofilms contain poly-GlcNAc and contribute to antibiotic resistance. *Front. Microbiol.* 11:585524. 10.3389/fmicb.2020.585524 33193233 PMC7652822

[B38] Deatherage B. L. Lara J. C. Bergsbaken T. Rassoulian Barrett S. L. Lara S. Cookson B. T. (2009). Biogenesis of bacterial membrane vesicles. *Mol. Microbiol.* 72 1395–1407. 10.1111/j.1365-2958.2009.06731.x 19432795 PMC2745257

[B39] Delekta P. C. Shook J. C. Lydic T. A. Mulks M. H. Hammer N. D. (2018). *Staphylococcus aureus* utilizes host-derived lipoprotein particles as sources of fatty acids. *J. Bacteriol.* 200:e00728-17. 10.1128/JB.00728-17 29581406 PMC5952394

[B40] Desvaux M. Hebraud M. Talon R. Henderson I. R. (2009). Secretion and subcellular localizations of bacterial proteins: a semantic awareness issue. *Trends Microbiol.* 17 139–145. 10.1016/j.tim.2009.01.004 19299134

[B41] Deutscher J. Ake F. M. Derkaoui M. Zebre A. C. Cao T. N. Bouraoui H. (2014). The bacterial phosphoenolpyruvate:carbohydrate phosphotransferase system: regulation by protein phosphorylation and phosphorylation-dependent protein-protein interactions. *Microbiol. Mol. Biol. Rev.* 78 231–256. 10.1128/MMBR.00001-14 24847021 PMC4054256

[B42] Diedershagen M. Overbeck S. Arlt S. Plumakers B. Lintges M. Rink L. (2007). *Mycoplasma arthritidis*-derived superantigen (MAM) displays DNase activity. *FEMS Immunol. Med. Microbiol.* 49 266–271. 10.1111/j.1574-695X.2006.00189.x 17328760

[B43] Djordjevic S. P. Cordwell S. J. Djordjevic M. A. Wilton J. Minion F. C. (2004). Proteolytic processing of the *Mycoplasma hyopneumoniae* cilium adhesin. *Infect. Immun.* 72 2791–2802. 10.1128/iai.72.5.2791-2802.2004 15102789 PMC387856

[B44] Domermuth C. H. Nielsen M. H. Freundt E. A. Birch-Andersen A. (1964). Ultrastructure of *Mycoplasma* species. *J. Bacteriol.* 88 727–744. 10.1128/JB.88.3.727-744.1964 14208513 PMC277372

[B45] Dwivedi P. Alam S. I. Tomar R. S. (2016). Secretome, surfome and immunome: emerging approaches for the discovery of new vaccine candidates against bacterial infections. *World J. Microbiol. Biotechnol.* 32:155. 10.1007/s11274-016-2107-3 27465855

[B46] Eng J. Froholm O. (1971). Immune electron microscopy of not cell-bound antigen of *Mycoplasma pneumoniae*. *Acta Pathol. Microbiol. Scand. B Microbiol. Immunol.* 79 759–763. 10.1111/j.1699-0463.1971.tb00108.x 5290405

[B47] Ganter S. Miotello G. Manso-Silvan L. Armengaud J. Tardy F. Gaurivaud P. (2019). Proteases as secreted exoproteins in mycoplasmas from ruminant lungs and their impact on surface-exposed proteins. *Appl. Environ. Microbiol.* 85:e01439-19. 10.1128/AEM.01439-19 31540994 PMC6856322

[B48] Gaurivaud P. Baranowski E. Pau-Roblot C. Sagne E. Citti C. Tardy F. (2016). *Mycoplasma agalactiae* secretion of beta-(1->6)-glucan, a rare polysaccharide in prokaryotes, is governed by high-frequency phase variation. *Appl. Environ. Microbiol.* 82 3370–3383. 10.1128/AEM.00274-16 27037120 PMC4959233

[B49] Gaurivaud P. Ganter S. Villard A. Manso-Silvan L. Chevret D. Boule C. (2018). Mycoplasmas are no exception to extracellular vesicles release: revisiting old concepts. *PLoS One* 13:e0208160. 10.1371/journal.pone.0208160 30485365 PMC6261642

[B50] Gaurivaud P. Lakhdar L. Le Grand D. Poumarat F. Tardy F. (2014). Comparison of *in vivo* and *in vitro* properties of capsulated and noncapsulated variants of *Mycoplasma mycoides* subsp. *mycoides* strain Afade: a potential new insight into the biology of contagious bovine pleuropneumonia. *FEMS Microbiol. Lett.* 359 42–49. 10.1111/1574-6968.12579 25123820

[B51] Gaurivaud P. Persson A. Grand D. L. Westberg J. Solsona M. Johansson K. E. (2004). Variability of a glucose phosphotransferase system permease in *Mycoplasma mycoides* subsp. *mycoides* Small Colony. *Microbiology (Reading)* 150(Pt. 12) 4009–4022. 10.1099/mic.0.27247-0 15583154

[B52] Gonzales J. Chakraborty T. Romero M. Mraheil M. A. Kutlar A. Pace B. (2021). *Streptococcus pneumoniae* and its virulence factors H2O2 and pneumolysin are potent mediators of the acute chest syndrome in sickle cell disease. *Toxins (Basel)* 13:157. 10.3390/toxins13020157 33671422 PMC7922783

[B53] Gourlay R. N. (1965). Antigenicity of *Mycoplasma mycoides*. II. Further studies on the precipitating antigens in the body fluids from cases of contagious bovine pleuropneumonia. *Res. Vet. Sci.* 6 1–8. 14281664

[B54] Grosshennig S. Ischebeck T. Gibhardt J. Busse J. Feussner I. Stulke J. (2016). Hydrogen sulfide is a novel potential virulence factor of *Mycoplasma pneumoniae*: characterization of the unusual cysteine desulfurase/desulfhydrase HapE. *Mol. Microbiol.* 100 42–54. 10.1111/mmi.13300 26711628

[B55] Grundel A. Jacobs E. Dumke R. (2016). Interactions of surface-displayed glycolytic enzymes of *Mycoplasma pneumoniae* with components of the human extracellular matrix. *Int. J. Med. Microbiol.* 306 675–685. 10.1016/j.ijmm.2016.09.001 27616280

[B56] Hagemann L. Grundel A. Jacobs E. Dumke R. (2017). The surface-displayed chaperones GroEL and DnaK of *Mycoplasma pneumoniae* interact with human plasminogen and components of the extracellular matrix. *Pathog. Dis.* 75:ftx017. 10.1093/femspd/ftx017 28204467

[B57] Hames C. Halbedel S. Hoppert M. Frey J. Stulke J. (2009). Glycerol metabolism is important for cytotoxicity of *Mycoplasma pneumoniae*. *J. Bacteriol.* 191 747–753. 10.1128/JB.01103-08 19028882 PMC2632104

[B58] Hernandez R. E. Gallegos-Monterrosa R. Coulthurst S. J. (2020). Type VI secretion system effector proteins: effective weapons for bacterial competitiveness. *Cell. Microbiol.* 22:e13241. 10.1111/cmi.13241 32592518

[B59] Herrero E. R. Slomka V. Bernaerts K. Boon N. Hernandez-Sanabria E. Passoni B. B. (2016). Antimicrobial effects of commensal oral species are regulated by environmental factors. *J. Dent.* 47 23–33. 10.1016/j.jdent.2016.02.007 26875613

[B60] Hopfe M. Hoffmann R. Henrich B. (2004). P80, the HinT interacting membrane protein, is a secreted antigen of *Mycoplasma hominis*. *BMC Microbiol.* 4:46. 10.1186/1471-2180-4-46 15579213 PMC539234

[B61] Houot L. Chang S. Pickering B. S. Absalon C. Watnick P. I. (2010). The phosphoenolpyruvate phosphotransferase system regulates *Vibrio cholerae* biofilm formation through multiple independent pathways. *J. Bacteriol.* 192 3055–3067. 10.1128/JB.00213-10 20400550 PMC2901703

[B62] Hudson J. R. Buttery S. Cottew G. S. (1967). Investigations into the influence of the galactan of *Mycoplasma mycoides* on experimental infection with that organism. *J. Pathol. Bacteriol.* 94 257–273. 10.1002/path.1700940204 4965241

[B63] Hummeler K. Tomassini N. Hayflick L. (1965). Ultrastructure of a *Mycoplasma* (*negroni*) isolated from human leukemia. *J. Bacteriol.* 90 517–523. 10.1128/JB.90.2.517-523.1965 14329468 PMC315673

[B64] Hwang M. H. Damte D. Cho M. H. Kim Y. H. Park S. C. (2010). Optimization of culture media of pathogenic *Mycoplasma hyopneumoniae* by a response surface methodology. *J. Vet. Sci.* 11 327–332. 10.4142/jvs.2010.11.4.327 21113102 PMC2998744

[B65] Jarocki V. M. Raymond B. B. A. Tacchi J. L. Padula M. P. Djordjevic S. P. (2019). *Mycoplasma hyopneumoniae* surface-associated proteases cleave bradykinin, substance P, neurokinin A and neuropeptide Y. *Sci. Rep.* 9:14585. 10.1038/s41598-019-51116-w 31601981 PMC6787215

[B66] Jarocki V. M. Santos J. Tacchi J. L. Raymond B. B. Deutscher A. T. Jenkins C. (2015). MHJ_0461 is a multifunctional leucine aminopeptidase on the surface of *Mycoplasma hyopneumoniae*. *Open Biol.* 5:140175. 10.1098/rsob.140175 25589579 PMC4313372

[B67] Jiang L. Schinkel M. van Essen M. Schiffelers R. M. (2019). Bacterial membrane vesicles as promising vaccine candidates. *Eur. J. Pharm. Biopharm.* 145 1–6. 10.1016/j.ejpb.2019.09.021 31560955

[B68] Jiang Z. Li S. Zhu C. Zhou R. Leung P. H. M. (2021). *Mycoplasma pneumoniae* infections: pathogenesis and vaccine development. *Pathogens* 10:119. 10.3390/pathogens10020119 33503845 PMC7911756

[B69] Jordan D. S. Daubenspeck J. M. Dybvig K. (2013). Rhamnose biosynthesis in mycoplasmas requires precursor glycans larger than monosaccharide. *Mol. Microbiol.* 89 918–928. 10.1111/mmi.12320 23826905 PMC3771393

[B70] Josi C. Burki S. Vidal S. Dordet-Frisoni E. Citti C. Falquet L. (2019). Large-scale analysis of the *Mycoplasma bovis* genome identified non-essential, adhesion- and virulence-related genes. *Front. Microbiol.* 10:2085. 10.3389/fmicb.2019.02085 31572317 PMC6753880

[B71] Khan L. A. Miles R. J. Nicholas R. A. (2005). Hydrogen peroxide production by *Mycoplasma bovis* and *Mycoplasma agalactiae* and effect of *in vitro* passage on a *Mycoplasma bovis* strain producing high levels of H2O2. *Vet. Res. Commun.* 29 181–188. 10.1023/b:verc.0000047506.04096.06 15736853

[B72] Kim J. H. Lee J. Park J. Gho Y. S. (2015). Gram-negative and Gram-positive bacterial extracellular vesicles. *Semin. Cell Dev. Biol.* 40 97–104. 10.1016/j.semcdb.2015.02.006 25704309

[B73] Klimentova J. Stulik J. (2015). Methods of isolation and purification of outer membrane vesicles from gram-negative bacteria. *Microbiol. Res.* 170 1–9. 10.1016/j.micres.2014.09.006 25458555

[B74] Konoshenko M. Y. Lekchnov E. A. Vlassov A. V. Laktionov P. P. (2018). Isolation of extracellular vesicles: general methodologies and latest trends. *Biomed Res. Int.* 2018:8545347. 10.1155/2018/8545347 29662902 PMC5831698

[B75] Kornilov R. Puhka M. Mannerstrom B. Hiidenmaa H. Peltoniemi H. Siljander P. (2018). Efficient ultrafiltration-based protocol to deplete extracellular vesicles from fetal bovine serum. *J. Extracell. Vesicles* 7:1422674. 10.1080/20013078.2017.1422674 29410778 PMC5795649

[B76] Kurotchkin T. J. (1937). Specific carbohydrate from *Asterococcus mycoides* for serologic tests of bovine pleuropneumonia. *Proc. Soc. Exp. Biol. Med.* 37 21–22.

[B77] Kurotchkin T. J. Benaradsky C. V. (1938). Serological diagnosis of bovine pleuropneumonia through the use of the specific carbohydrate of *Asterococcus mycoides*. *Chin. Med. J.* Suppl. 2 269–278.

[B78] Lahteenmaki K. Edelman S. Korhonen T. K. (2005). Bacterial metastasis: the host plasminogen system in bacterial invasion. *Trends Microbiol.* 13 79–85. 10.1016/j.tim.2004.12.003 15680767

[B79] Le Carrou J. Laurentie M. Kobisch M. Gautier-Bouchardon A. V. (2006). Persistence of *Mycoplasma hyopneumoniae* in experimentally infected pigs after marbofloxacin treatment and detection of mutations in the parC gene. *Antimicrob. Agents Chemother.* 50 1959–1966. 10.1128/AAC.01527-05 16723552 PMC1479153

[B80] Leal Zimmer F. M. A. Paes J. A. Zaha A. Ferreira H. B. (2020). Pathogenicity and virulence of *Mycoplasma hyopneumoniae*. *Virulence* 11 1600–1622. 10.1080/21505594.2020.1842659 33289597 PMC7733983

[B81] Leal Zimmer F. Paludo G. P. Moura H. Barr J. R. Ferreira H. B. (2019). Differential secretome profiling of a swine tracheal cell line infected with mycoplasmas of the swine respiratory tract. *J. Proteomics* 192 147–159. 10.1016/j.jprot.2018.08.018 30176387

[B82] Li P. Zhang Y. Li X. Zhou W. Li X. Jiang F. (2019). *Mycoplasma hyopneumoniae* Mhp597 is a cytotoxicity, inflammation and immunosuppression associated nuclease. *Vet. Microbiol.* 235 53–62. 10.1016/j.vetmic.2019.05.011 31282379

[B83] Limoli D. H. Jones C. J. Wozniak D. J. (2015). Bacterial extracellular polysaccharides in biofilm formation and function. *Microbiol. Spectr.* 3. 10.1128/microbiolspec.MB-0011-2014 26185074 PMC4657554

[B84] Lin J. Zhang W. Cheng J. Yang X. Zhu K. Wang Y. (2017). A *Pseudomonas* T6SS effector recruits PQS-containing outer membrane vesicles for iron acquisition. *Nat. Commun.* 8:14888. 10.1038/ncomms14888 28348410 PMC5379069

[B85] Llobet E. Tomas J. M. Bengoechea J. A. (2008). Capsule polysaccharide is a bacterial decoy for antimicrobial peptides. *Microbiology (Reading)* 154(Pt. 12) 3877–3886. 10.1099/mic.0.2008/022301-0 19047754

[B86] Lloyd L. C. (1966). Tissue necrosis produced by *Mycoplasma mycoides* in intraperitoneal diffusion chambers. *Pathol. Bacteriol.* 92 225–229. 10.1002/path.1700920125 5956261

[B87] Machado L. Paes J. A. Souza Dos Santos P. Ferreira H. B. (2020). Evidences of differential endoproteolytic processing on the surfaces of *Mycoplasma hyopneumoniae* and *Mycoplasma flocculare*. *Microb. Pathog.* 140:103958. 10.1016/j.micpath.2019.103958 31899326

[B88] Madsen M. L. Nettleton D. Thacker E. L. Minion F. C. (2006). Transcriptional profiling of *Mycoplasma hyopneumoniae* during iron depletion using microarrays. *Microbiology (Reading)* 152(Pt. 4) 937–944. 10.1099/mic.0.28674-0 16549658

[B89] Marois C. Le Carrou J. Kobisch M. Gautier-Bouchardon A. V. (2007). Isolation of *Mycoplasma hyopneumoniae* from different sampling sites in experimentally infected and contact SPF piglets. *Vet. Microbiol.* 120 96–104. 10.1016/j.vetmic.2006.10.015 17116374

[B90] Martinez-Torro C. Torres-Puig S. Monge M. Sanchez-Alba L. Gonzalez-Martin M. Marcos-Silva M. (2020). Transcriptional response to metal starvation in the emerging pathogen *Mycoplasma genitalium* is mediated by Fur-dependent and -independent regulatory pathways. *Emerg. Microbes Infect.* 9 5–19. 10.1080/22221751.2019.1700762 31859607 PMC6968530

[B91] McGowin C. L. Popov V. L. Pyles R. B. (2009). Intracellular *Mycoplasma genitalium* infection of human vaginal and cervical epithelial cells elicits distinct patterns of inflammatory cytokine secretion and provides a possible survival niche against macrophage-mediated killing. *BMC Microbiol.* 9:139. 10.1186/1471-2180-9-139 19602269 PMC2717097

[B92] McMillan H. M. Kuehn M. J. (2021). The extracellular vesicle generation paradox: a bacterial point of view. *EMBO J.* 40:e108174. 10.15252/embj.2021108174 34636061 PMC8561641

[B93] Minion F. C. Jarvill-Taylor K. J. Billings D. E. Tigges E. (1993). Membrane-associated nuclease activities in mycoplasmas. *J. Bacteriol.* 175 7842–7847. 10.1128/jb.175.24.7842-7847.1993 8253673 PMC206960

[B94] Monteiro R. Chafsey I. Ageorges V. Leroy S. Chambon C. Hebraud M. (2021). The Secretome landscape of *Escherichia coli* O157:H7: deciphering the cell-surface, outer membrane vesicle and extracellular subproteomes. *J. Proteomics* 232:104025. 10.1016/j.jprot.2020.104025 33160105

[B95] Mu H. H. Sawitzke A. D. Cole B. C. (2000). Modulation of cytokine profiles by the *Mycoplasma* superantigen *Mycoplasma arthritidis* mitogen parallels susceptibility to arthritis induced by *M. arthritidis*. *Infect. Immun.* 68 1142–1149. 10.1128/iai.68.3.1142-1149.2000 10678918 PMC97259

[B96] Nguyen M. T. Peisl L. Barletta F. Luqman A. Gotz F. (2018). Toll-like receptor 2 and lipoprotein-like lipoproteins enhance *Staphylococcus aureus* invasion in epithelial cells. *Infect. Immun.* 86:e00343-18. 10.1128/IAI.00343-18 29844243 PMC6056871

[B97] Nocard, and Roux (1898). The microbe of pleuropneumonia. *Rev. Infect. Dis.* 12 354–358. 10.1093/clinids/12.2.354 2184501

[B98] Olaya-Abril A. Gonzalez-Reyes J. A. Rodriguez-Ortega M. J. (2021). Approaching *in vivo* models of pneumococcus-host interaction: insights into surface proteins, capsule production, and extracellular vesicles. *Pathogens* 10:1098. 10.3390/pathogens10091098 34578131 PMC8471892

[B99] Orench-Rivera N. Kuehn M. J. (2021). Differential packaging into outer membrane vesicles upon oxidative stress reveals a general mechanism for cargo selectivity. *Front. Microbiol.* 12:561863. 10.3389/fmicb.2021.561863 34276573 PMC8284480

[B100] Paes J. A. Lorenzatto K. R. de Moraes S. N. Moura H. Barr J. R. Ferreira H. B. (2017). Secretomes of *Mycoplasma hyopneumoniae* and *Mycoplasma flocculare* reveal differences associated to pathogenesis. *J. Proteomics* 154 69–77. 10.1016/j.jprot.2016.12.002 28003119

[B101] Pilo P. Vilei E. M. Peterhans E. Bonvin-Klotz L. Stoffel M. H. Dobbelaere D. (2005). A metabolic enzyme as a primary virulence factor of *Mycoplasma mycoides* subsp. *mycoides* small colony. *J. Bacteriol.* 187 6824–6831. 10.1128/JB.187.19.6824-6831.2005 16166545 PMC1251598

[B102] Plackett P. Buttery S. H. (1958). A galactan from *Mycoplasma mycoides*. *Nature* 182 1236–1237. 10.1038/1821236a0 13590288

[B103] Plackett P. Buttery S. H. (1964). A galactofuranose disaccharide from the galactan of *Mycoplasma mycoides*. *Biochem. J.* 90 201–205. 10.1042/bj0900201 5832292 PMC1202544

[B104] Plackett P. Buttery S. Cottew G. (1963). “Carbohydrates of some *Mycoplasma* strains,” in *Recent Progress in Microbiology VIII*, ed. Gibbons N. E. (Toronto, ON: University of Toronto Press), 533.

[B105] Prados-Rosales R. Weinrick B. C. Pique D. G. Jacobs W. R. Jr. Casadevall A. Rodriguez G. M. (2014). Role for *Mycobacterium tuberculosis* membrane vesicles in iron acquisition. *J. Bacteriol.* 196 1250–1256. 10.1128/JB.01090-13 24415729 PMC3957709

[B106] Raymond B. B. A. Turnbull L. Jenkins C. Madhkoor R. Schleicher I. Uphoff C. C. (2018b). *Mycoplasma hyopneumoniae* resides intracellularly within porcine epithelial cells. *Sci. Rep.* 8:17697. 10.1038/s41598-018-36054-3 30523267 PMC6283846

[B107] Raymond B. B. A. Jenkins C. Turnbull L. Whitchurch C. B. Djordjevic S. P. (2018a). Extracellular DNA release from the genome-reduced pathogen *Mycoplasma hyopneumoniae* is essential for biofilm formation on abiotic surfaces. *Sci. Rep.* 8:10373. 10.1038/s41598-018-28678-2 29991767 PMC6039474

[B108] Raymond B. B. Tacchi J. L. Jarocki V. M. Minion F. C. Padula M. P. Djordjevic S. P. (2013). P159 from *Mycoplasma hyopneumoniae* binds porcine cilia and heparin and is cleaved in a manner akin to ectodomain shedding. *J. Proteome Res.* 12 5891–5903. 10.1021/pr400903s 24195521

[B109] Razin S. Hasin M. Ne’eman Z. Rottem S. (1973). Isolation, chemical composition, and ultrastructural features of the cell membrane of the mycoplasma-like organism *Spiroplasma citri*. *J. Bacteriol.* 116 1421–1435. 10.1128/jb.116.3.1421-1435.1973 4127633 PMC246502

[B110] Razin S. Yogev D. Naot Y. (1998). Molecular biology and pathogenicity of mycoplasmas. *Microbiol. Mol. Biol. Rev.* 62 1094–1156. 10.1128/MMBR.62.4.1094-1156.1998 9841667 PMC98941

[B111] Rebollo Couto M. S. Klein C. S. Voss-Rech D. Terenzi H. (2012). Extracellular proteins of *Mycoplasma synoviae*. *ISRN Vet. Sci.* 2012:802308. 10.5402/2012/802308 23762591 PMC3671734

[B112] Robinson M. W. Buchtmann K. A. Jenkins C. Tacchi J. L. Raymond B. B. To J. (2013). MHJ_0125 is an M42 glutamyl aminopeptidase that moonlights as a multifunctional adhesin on the surface of *Mycoplasma hyopneumoniae*. *Open Biol.* 3:130017. 10.1098/rsob.130017 23594879 PMC3718333

[B113] Rojas E. R. Huang K. C. (2018). Regulation of microbial growth by turgor pressure. *Curr. Opin Microbiol.* 42 62–70. 10.1016/j.mib.2017.10.015 29125939

[B114] Sacchini F. Luciani M. Salini R. Scacchia M. Pini A. Lelli R. (2012). Plasma levels of TNF-alpha, IFN-gamma, IL-4 and IL-10 during a course of experimental contagious bovine pleuropneumonia. *BMC Vet. Res.* 8:44. 10.1186/1746-6148-8-44 22533922 PMC3378467

[B115] Schieck E. Lartigue C. Frey J. Vozza N. Hegermann J. Miller R. A. (2016). Galactofuranose in *Mycoplasma mycoides* is important for membrane integrity and conceals Adhesins but does not contribute to serum resistance. *Mol. Microbiol.* 99 55–70. 10.1111/mmi.13213 26354009

[B116] Schmid J. (2018). Recent insights in microbial exopolysaccharide biosynthesis and engineering strategies. *Curr. Opin. Biotechnol.* 53 130–136. 10.1016/j.copbio.2018.01.005 29367163

[B117] Schmidt J. A. Browning G. F. Markham P. F. (2004). *Mycoplasma hyopneumoniae* p65 surface lipoprotein is a lipolytic enzyme with a preference for shorter-chain fatty acids. *J. Bacteriol.* 186 5790–5798. 10.1128/JB.186.17.5790-5798.2004 15317784 PMC516823

[B118] Seymour L. M. Jenkins C. Deutscher A. T. Raymond B. B. Padula M. P. Tacchi J. L. (2012). Mhp182 (P102) binds fibronectin and contributes to the recruitment of plasmin(ogen) to the *Mycoplasma hyopneumoniae* cell surface. *Cell. Microbiol.* 14 81–94. 10.1111/j.1462-5822.2011.01702.x 21951786

[B119] Sharma A. K. Dhasmana N. Dubey N. Kumar N. Gangwal A. Gupta M. (2017). Bacterial virulence factors: secreted for survival. *Indian J. Microbiol.* 57 1–10. 10.1007/s12088-016-0625-1 28148975 PMC5243249

[B120] Sharma S. Markham P. F. Browning G. F. (2014). Genes found essential in other mycoplasmas are dispensable in *Mycoplasma bovis*. *PLoS One* 9:e97100. 10.1371/journal.pone.0097100 24897538 PMC4045577

[B121] Sharma S. Tivendale K. A. Markham P. F. Browning G. F. (2015). Disruption of the membrane nuclease gene (MBOVPG45_0215) of *Mycoplasma bovis* greatly reduces cellular nuclease activity. *J. Bacteriol.* 197 1549–1558. 10.1128/JB.00034-15 25691526 PMC4403647

[B122] Shaw B. M. Daubenspeck J. M. Simmons W. L. Dybvig K. (2013). EPS-I polysaccharide protects *Mycoplasma pulmonis* from phagocytosis. *FEMS Microbiol. Lett.* 338 155–160. 10.1111/1574-6968.12048 23190331 PMC3535568

[B123] Shin J. Rhim J. Kwon Y. Choi S. Y. Shin S. Ha C. W. (2019). Comparative analysis of differentially secreted proteins in serum-free and serum-containing media by using BONCAT and pulsed SILAC. *Sci. Rep.* 9:3096. 10.1038/s41598-019-39650-z 30816242 PMC6395664

[B124] Siegl C. Rudel T. (2015). Modulation of p53 during bacterial infections. *Nat. Rev. Microbiol.* 13 741–748. 10.1038/nrmicro3537 26548915

[B125] Simmons W. L. Daubenspeck J. M. Osborne J. D. Balish M. F. Waites K. B. Dybvig K. (2013). Type 1 and type 2 strains of *Mycoplasma pneumoniae* form different biofilms. *Microbiology (Reading)* 159(Pt. 4) 737–747. 10.1099/mic.0.064782-0 23412845 PMC4036059

[B126] Song Z. Li Y. Liu Y. Xin J. Zou X. Sun W. (2012). alpha-Enolase, an adhesion-related factor of *Mycoplasma bovis*. *PLoS One* 7:e38836. 10.1371/journal.pone.0038836 22719960 PMC3374825

[B127] Staats C. C. Boldo J. T. Broetto L. Vainstein M. H. Schrank A. (2007). Comparative genome analysis of proteases, oligopeptide uptake and secretion systems in *Mycoplasma* spp. *Genet. Mol. Biol.* 30 225–229.

[B128] Stentz R. Carvalho A. L. Jones E. J. Carding S. R. (2018). Fantastic voyage: the journey of intestinal microbiota-derived microvesicles through the body. *Biochem. Soc. Trans.* 46 1021–1027. 10.1042/BST20180114 30154095 PMC6195637

[B129] Stentz R. Miquel-Clopes A. Carding S. R. (2022). Production, isolation, and characterization of bioengineered bacterial extracellular membrane vesicles derived from *Bacteroides thetaiotaomicron* and their use in vaccine development. *Methods Mol. Biol.* 2414 171–190. 10.1007/978-1-0716-1900-1_11 34784038

[B130] Sukumaran A. Woroszchuk E. Ross T. Geddes-McAlister J. (2021). Proteomics of host-bacterial interactions: new insights from dual perspectives. *Can. J. Microbiol.* 67 213–225. 10.1139/cjm-2020-0324 33027598

[B131] Sutherland I. W. (1972). Bacterial exopolysaccharides. *Adv. Microb. Physiol.* 8 143–213. 10.1016/s0065-2911(08)60190-3 4581483

[B132] Sutherland I. W. (1985). Biosynthesis and composition of Gram-negative bacterial extracellular and wall polysaccharides. *Annu. Rev. Microbiol.* 39 243–270. 10.1146/annurev.mi.39.100185.001331 3904602

[B133] Tacchi J. L. Raymond B. B. Haynes P. A. Berry I. J. Widjaja M. Bogema D. R. (2016). Post-translational processing targets functionally diverse proteins in *Mycoplasma hyopneumoniae*. *Open Biol.* 6:150210. 10.1098/rsob.150210 26865024 PMC4772806

[B134] Tam K. Torres V. J. (2019). *Staphylococcus aureus* secreted toxins and extracellular enzymes. *Microbiol. Spectr.* 7. 10.1128/microbiolspec.GPP3-0039-2018 30873936 PMC6422052

[B135] Thery C. Witwer K. W. Aikawa E. Alcaraz M. J. Anderson J. D. Andriantsitohaina R. (2018). Minimal information for studies of extracellular vesicles 2018 (MISEV2018): a position statement of the International Society for Extracellular Vesicles and update of the MISEV2014 guidelines. *J. Extracell. Vesicles* 7:1535750. 10.1080/20013078.2018.1535750 30637094 PMC6322352

[B136] Tjalsma H. Bolhuis A. Jongbloed J. D. Bron S. van Dijl J. M. (2000). Signal peptide-dependent protein transport in *Bacillus subtilis*: a genome-based survey of the secretome. *Microbiol. Mol. Biol. Rev.* 64 515–547. 10.1128/MMBR.64.3.515-547.2000 10974125 PMC99003

[B137] Tommassen J. Arenas J. (2017). Biological functions of the secretome of *Neisseria meningitidis*. *Front. Cell. Infect. Microbiol.* 7:256. 10.3389/fcimb.2017.00256 28670572 PMC5472700

[B138] Totte P. Puech C. Rodrigues V. Bertin C. Manso-Silvan L. Thiaucourt F. (2015). Free exopolysaccharide from *Mycoplasma mycoides* subsp. *mycoides* possesses anti-inflammatory properties. *Vet. Res.* 46:122. 10.1186/s13567-015-0252-6 26490663 PMC4618858

[B139] Tourtellotte M. E. Morowitz H. J. Kasimer P. (1964). Defined medium for *Mycoplasma laidlawii*. *J. Bacteriol.* 88 11–15. 10.1128/JB.88.1.11-15.1964 14197875 PMC277248

[B140] Toyofuku M. Nomura N. Eberl L. (2019). Types and origins of bacterial membrane vesicles. *Nat. Rev. Microbiol.* 17 13–24. 10.1038/s41579-018-0112-2 30397270

[B141] Turnbull L. Toyofuku M. Hynen A. L. Kurosawa M. Pessi G. Petty N. K. (2016). Explosive cell lysis as a mechanism for the biogenesis of bacterial membrane vesicles and biofilms. *Nat. Commun.* 7:11220. 10.1038/ncomms11220 27075392 PMC4834629

[B142] Voros A. DeLongchamp J. Saleh M. T. (2015). The secretome of *Mycoplasma capricolum* subsp. *capricolum* in neutral and acidic media. *J. Proteomics Bioinformatics* 8 155–163.

[B143] Voros A. Dunnett A. Leduc L. G. Saleh M. T. (2009). Depleting proteins from the growth medium of *Mycoplasma capricolum* unmasks bacterium-derived enzymatic activities. *Vet. Microbiol.* 138 384–389. 10.1016/j.vetmic.2009.04.012 19446411

[B144] Wang G. Chen H. Xia Y. Cui J. Gu Z. Song Y. (2013). How are the non-classically secreted bacterial proteins released into the extracellular milieu? *Curr. Microbiol.* 67 688–695. 10.1007/s00284-013-0422-6 23963513

[B145] Wang J. Li Y. Pan L. Li J. Yu Y. Liu B. (2021). Glyceraldehyde-3-phosphate dehydrogenase (GAPDH) moonlights as an adhesin in *Mycoplasma hyorhinis* adhesion to epithelial cells as well as a plasminogen receptor mediating extracellular matrix degradation. *Vet. Res.* 52:80. 10.1186/s13567-021-00952-8 34082810 PMC8173509

[B146] Wawegama N. K. Browning G. F. Kanci A. Marenda M. S. Markham P. F. (2014). Development of a recombinant protein-based enzyme-linked immunosorbent assay for diagnosis of *Mycoplasma bovis* infection in cattle. *Clin. Vaccine Immunol.* 21 196–202. 10.1128/CVI.00670-13 24334686 PMC3910936

[B147] Whitney J. C. Howell P. L. (2013). Synthase-dependent exopolysaccharide secretion in Gram-negative bacteria. *Trends Microbiol.* 21 63–72. 10.1016/j.tim.2012.10.001 23117123 PMC4113494

[B148] Widjaja M. Harvey K. L. Hagemann L. Berry I. J. Jarocki V. M. Raymond B. B. A. (2017). Elongation factor Tu is a multifunctional and processed moonlighting protein. *Sci. Rep.* 7:11227. 10.1038/s41598-017-10644-z 28894125 PMC5593925

[B149] Woodward L. Naismith J. H. (2016). Bacterial polysaccharide synthesis and export. *Curr. Opin. Struct. Biol.* 40 81–88. 10.1016/j.sbi.2016.07.016 27544430

[B150] Woolley L. K. Fell S. Gonsalves J. R. Walker M. J. Djordjevic S. P. Jenkins C. (2012). Evaluation of clinical, histological and immunological changes and qPCR detection of *Mycoplasma hyopneumoniae* in tissues during the early stages of mycoplasmal pneumonia in pigs after experimental challenge with two field isolates. *Vet. Microbiol.* 161 186–195. 10.1016/j.vetmic.2012.07.025 22863144

[B151] Yamamoto T. Kida Y. Sakamoto Y. Kuwano K. (2017). Mpn491, a secreted nuclease of *Mycoplasma pneumoniae*, plays a critical role in evading killing by neutrophil extracellular traps. *Cell. Microbiol.* 19:e12666. 10.1111/cmi.12666 27603754

[B152] Yavlovich A. Katzenell A. Tarshis M. Higazi A. A. Rottem S. (2004a). *Mycoplasma fermentans* binds to and invades HeLa cells: involvement of plasminogen and urokinase. *Infect. Immun.* 72 5004–5011. 10.1128/IAI.72.9.5004-5011.2004 15321992 PMC517474

[B153] Yavlovich A. Tarshis M. Rottem S. (2004b). Internalization and intracellular survival of *Mycoplasma pneumoniae* by non-phagocytic cells. *FEMS Microbiol. Lett.* 233 241–246. 10.1016/j.femsle.2004.02.016 15063492

[B154] Yiwen C. Yueyue W. Lianmei Q. Cuiming Z. Xiaoxing Y. (2021). Infection strategies of mycoplasmas: unraveling the panoply of virulence factors. *Virulence* 12 788–817. 10.1080/21505594.2021.1889813 33704021 PMC7954426

[B155] Yus E. Maier T. Michalodimitrakis K. van Noort V. Yamada T. Chen W. H. (2009). Impact of genome reduction on bacterial metabolism and its regulation. *Science* 326 1263–1268. 10.1126/science.1177263 19965476

[B156] Zella D. Curreli S. Benedetti F. Krishnan S. Cocchi F. Latinovic O. S. (2018). Mycoplasma promotes malignant transformation in vivo, and its DnaK, a bacterial chaperone protein, has broad oncogenic properties. *Proc. Natl. Acad. Sci. U.S.A.* 115 E12005–E12014. 10.1073/pnas.1815660115 30509983 PMC6304983

[B157] Zhang H. Hu G. Lu D. Zhao G. Zhang Y. Zubair M. (2021). Comparative secretome analyses of *Mycoplasma bovis* virulent and attenuated strains revealed MbovP0145 as a promising diagnostic biomarker. *Front. Vet. Sci.* 8:666769. 10.3389/fvets.2021.666769 34222397 PMC8249566

[B158] Zhang H. Zhao G. Guo Y. Menghwar H. Chen Y. Chen H. (2016). *Mycoplasma bovis* MBOV_RS02825 encodes a secretory nuclease associated with cytotoxicity. *Int. J. Mol. Sci.* 17:628. 10.3390/ijms17050628 27136546 PMC4881454

[B159] Zhao G. Zhang H. Chen X. Zhu X. Guo Y. He C. (2017). *Mycoplasma bovis* NADH oxidase functions as both a NADH oxidizing and O2 reducing enzyme and an adhesin. *Sci. Rep.* 7:44. 10.1038/s41598-017-00121-y 28246386 PMC5427908

[B160] Zhao G. Zhu X. Zhang H. Chen Y. Schieck E. Hu C. (2021). Novel secreted protein of *Mycoplasma bovis* MbovP280 induces macrophage apoptosis through CRYAB. *Front. Immunol.* 12:619362. 10.3389/fimmu.2021.619362 33659004 PMC7917047

[B161] Zhu X. Dordet-Frisoni E. Gillard L. Ba A. Hygonenq M. C. Sagne E. (2019). Extracellular DNA: a nutritional trigger of *Mycoplasma bovis* cytotoxicity. *Front. Microbiol.* 10:2753. 10.3389/fmicb.2019.02753 31849895 PMC6895004

[B162] Zubair M. Khan F. A. Menghwar H. Faisal M. Ashraf M. Rasheed M. A. (2020a). Progresses on bacterial secretomes enlighten research on *Mycoplasma* secretome. *Microb. Pathog.* 144:104160. 10.1016/j.micpath.2020.104160 32194181

[B163] Zubair M. Muhamed S. A. Khan F. A. Zhao G. Menghwar H. Faisal M. (2020b). Identification of 60 secreted proteins for *Mycoplasma bovis* with secretome assay. *Microb. Pathog.* 143:104135. 10.1016/j.micpath.2020.104135 32165330

